# Reprogramming reactive glia into interneurons reduces chronic seizure activity in a mouse model of mesial temporal lobe epilepsy

**DOI:** 10.1016/j.stem.2021.09.002

**Published:** 2021-12-02

**Authors:** Célia Lentini, Marie d’Orange, Nicolás Marichal, Marie-Madeleine Trottmann, Rory Vignoles, Louis Foucault, Charlotte Verrier, Céline Massera, Olivier Raineteau, Karl-Klaus Conzelmann, Sylvie Rival-Gervier, Antoine Depaulis, Benedikt Berninger, Christophe Heinrich

**Affiliations:** 1 Univ Lyon, Université Claude Bernard Lyon 1, Inserm, Stem Cell and Brain Research Institute U1208, 69500 Bron, France; 2 Centre for Developmental Neurobiology, Institute of Psychiatry, Psychology & Neuroscience, King’s College London, London SE1 1UL, UK; 3 MRC Centre for Neurodevelopmental Disorders, King’s College London, London SE1 1UL, UK; 4 Univ Grenoble Alpes, Inserm U1216, Grenoble Institut des Neurosciences, 38000 Grenoble, France; 5 Max von Pettenkofer-Institute Virology, Medical Faculty & Gene Center, Ludwig-Maximilians-University, 81377 Munich, Germany; 6 Univ Lyon, Université Claude Bernard Lyon 1, Inserm, INRAE, Stem Cell and Brain Research Institute U1208, CSC USC1361, 69500 Bron, France; 7 Institute of Physiological Chemistry, University Medical Center, Johannes Gutenberg University, 55128 Mainz, Germany

**Keywords:** direct lineage reprogramming, glia-to-neuron conversion, regeneration and repair in the nervous system, regenerative medicine, gene therapy, therapy-resistant epilepsy

## Abstract

Reprogramming brain-resident glial cells into clinically relevant induced neurons (iNs) is an emerging strategy toward replacing lost neurons and restoring lost brain functions. A fundamental question is now whether iNs can promote functional recovery in pathological contexts. We addressed this question in the context of therapy-resistant mesial temporal lobe epilepsy (MTLE), which is associated with hippocampal seizures and degeneration of hippocampal GABAergic interneurons. Using a MTLE mouse model, we show that retrovirus-driven expression of Ascl1 and Dlx2 in reactive hippocampal glia *in situ*, or in cortical astroglia grafted in the epileptic hippocampus, causes efficient reprogramming into iNs exhibiting hallmarks of interneurons. These induced interneurons functionally integrate into epileptic networks and establish GABAergic synapses onto dentate granule cells. MTLE mice with GABAergic iNs show a significant reduction in both the number and cumulative duration of spontaneous recurrent hippocampal seizures. Thus glia-to-neuron reprogramming is a potential disease-modifying strategy to reduce seizures in therapy-resistant epilepsy.

## Highlights

• Retroviruses target reactive hippocampal glia proliferating in a mouse model of mesial temporal lobe epilepsy
• Ascl1 and Dlx2 reprogram reactive glia into GABAergic interneurons in the epileptic hippocampus
• Induced interneurons establish GABAergic synapses onto dentate granule cells
• Induced interneurons reduce chronic epileptic activity in the hippocampus

## Graphical abstract

**Figure:**
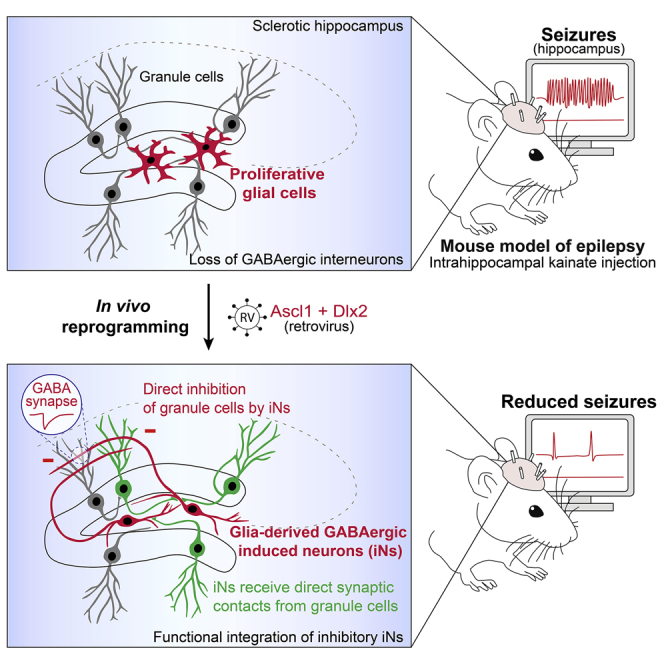

## Teaser

Mesial temporal lobe epilepsy belongs to treatment-refractory forms of human epilepsy. Lentini et al. show that reactive glia proliferating in the epileptic hippocampus can be reprogrammed into GABAergic induced neurons that reduce chronic seizure activity. This study uncovers glia-to-neuron reprogramming as a potential disease-modifying strategy to reduce intractable seizures.

## Introduction

The adult mammalian central nervous system lacks the intrinsic regenerative capacity to replace lost neurons and induce functional recovery. Regenerative medicine aims to replace damaged neurons by using cell-based strategies in order to restore lost functions and correct neurological deficits (Heinrich et al., 2015). An emerging approach toward this goal is to instruct fate conversion of brain-resident glial cells into clinically relevant induced neurons (iNs) by direct *in vivo* lineage reprogramming, which has been achieved by forced expression of neurogenic transcription factors (TFs) (Barker et al., 2018; Gascón et al., 2017; Vignoles et al., 2019). Since pioneering *in vitro* studies (Berninger et al., 2007; Heinrich et al., 2010), considerable progress has been made in instructing *in vivo* reprogramming of astroglia, NG2 glia, or microglia to generate functional iNs of various phenotypes within the adult mouse cortex (Gascón et al., 2016; Grande et al., 2013; Guo et al., 2014; Heinrich et al., 2014; Mattugini et al., 2019), striatum (Grande et al., 2013; Matsuda et al., 2019; Niu et al., 2013; Pereira et al., 2017; Rivetti di Val Cervo et al., 2017; Torper et al., 2013, 2015), and spinal cord (Su et al., 2014). Although glia-to-neuron reprogramming holds promise as a neuron-replacement strategy, a critical question is now whether iNs are endowed with the capability of promoting functional recovery in pathological contexts. Clinically effective reprogramming will require that iNs functionally and stably integrate within diseased circuits, not only receiving synaptic inputs from endogenous neurons but also selectively sending their axons onto target neurons to ultimately restore lost synaptic transmission and elicit a therapeutic response.

In the present study, we addressed these questions in the context of mesial temporal lobe epilepsy with hippocampal sclerosis (MTLE-HS), a well-characterized epileptic syndrome, which is the most common form of focal epilepsy and among the most treatment-refractory forms of human epilepsy (Engel, 2001). MTLE-HS is characterized by recurrence of focal dyscognitive seizures that originate in the sclerotic hippocampus or its adjacent mesial temporal structures, do not involve convulsions, and are typically resistant to antiepileptic drugs (Cendes et al., 2014; Engel, 2001). The histopathological features of hippocampal sclerosis most commonly described in MTLE-HS patients encompass severe neuronal loss predominantly in CA1 and CA4 hippocampal subfields as well as reactive gliosis (Blümcke et al., 2013b). Dispersion of granule cells (GCs) (Blümcke et al., 2013b) and mossy fiber sprouting (Sutula and Dudek, 2007) can also be observed. Importantly, a prominent feature is also a degeneration of hippocampal GABAergic interneurons of various subtypes (Andrioli et al., 2007; de Lanerolle et al., 1989; Robbins et al., 1991; Tóth et al., 2010; Wittner et al., 2005), which is associated with decreased synaptic inhibition of dentate GCs (Williamson et al., 1999). Accumulating evidence in animal models supports the notion that GABA neuron loss can promote the epileptic state (Cobos et al., 2005; Drexel et al., 2017). This is further illustrated by seizure reduction in epilepsy models after grafting of medial ganglionic eminence progenitor cells generating GABAergic interneurons in recipient epileptic brains (Baraban et al., 2009; Cunningham et al., 2014; Hunt et al., 2013; Upadhya et al., 2019) or following recruitment of surviving endogenous interneurons (Cǎlin et al., 2018). Currently, although invasive resective surgery of the epileptogenic zone can offer potential seizure control, up to 40% of patients having surgery show early or late surgical failures (Blümcke et al., 2013a). There is therefore a crucial need for effective disease-modifying treatments to achieve seizure control in patients with MTLE-HS.

We hypothesized that regeneration of GABAergic neurons by *in vivo* lineage reprogramming of glial cells could represent an innovative approach to reduce seizures in MTLE-HS. Using a well-established mouse model of chronic MTLE-HS, we show that reactive glial cells proliferating within the sclerotic hippocampus, or cortical astroglia grafted into the epileptic hippocampus, can be reprogrammed by Ascl1 and Dlx2 to generate iNs that (1) acquire a GABAergic identity, (2) functionally integrate into epileptic networks by establishing GABAergic synapses on GCs, and (3) reduce spontaneous recurrent hippocampal seizures.

## Results

### Loss of hippocampal interneurons in a mouse model of MTLE-HS

To study the potentially beneficial effects of iNs in epilepsy, we employed a well-established mouse model of chronic MTLE-HS (referred to as MTLE-HS mice) that is obtained by intrahippocampal injection of kainate (KA) in adult mice and recapitulates most pathophysiological features of human MTLE-HS (Arabadzisz et al., 2005; Heinrich et al., 2011b; Löscher et al., 2020; Maroso et al., 2010; Riban et al., 2002). We selected this model as one used by the NIH/NINDS Epilepsy Therapy Screening Program to test the efficacy of new antiepileptic treatments given the poor responsiveness of seizures to various antiepileptic drugs (Löscher et al., 2020), thus mimicking non-convulsive drug-resistant seizures described in MTLE-HS patients. In line with previous studies (Arabadzisz et al., 2005; Heinrich et al., 2006, 2011b; Maroso et al., 2010; Riban et al., 2002), unilateral KA injection triggered in all mice an initial non-convulsive status epilepticus (SE) followed by a latent phase of 2 weeks corresponding to development of spontaneous recurrent seizures (i.e., epileptogenesis, Figure S1A). Subsequently, during the chronic phase, focal non-convulsive electrographic seizures repeatedly occurred in all mice in the injected hippocampus (Figure S1B) without overt behavioral changes. As previously reported, KA injection induced a typical pattern of hippocampal sclerosis characterized by extensive neuronal loss in the ipsilateral hilus and CA hippocampal subfields (Figure S1C), dispersion of dentate GCs (Figures S1C and S1D), and reactive gliosis (Figures S3D–S3F), as observed in MTLE-HS patients (i.e., HS ILAE (International League Against Epilepsy) Type 1; Blümcke et al., 2013b). Importantly, in line with previous work (Bouilleret et al., 2000), we observed a severe and early loss of GABAergic interneurons as evidenced by massive reduction in GFP+ inhibitory neurons all over the ipsilateral hippocampus at 5 days post-KA injection (dpKA; Figures S1E and S1F) in GAD67-GFP mice (Tamamaki et al., 2003). Given the dramatic loss of interneurons, here, we set out to test the hypothesis that reprogramming of reactive glia into interneurons could promote amelioration of focal seizure activity.

### *In vivo* reprogramming of cortical astroglia into GABAergic iNs in the MTLE-HS mouse hippocampus

**Figure 1:**
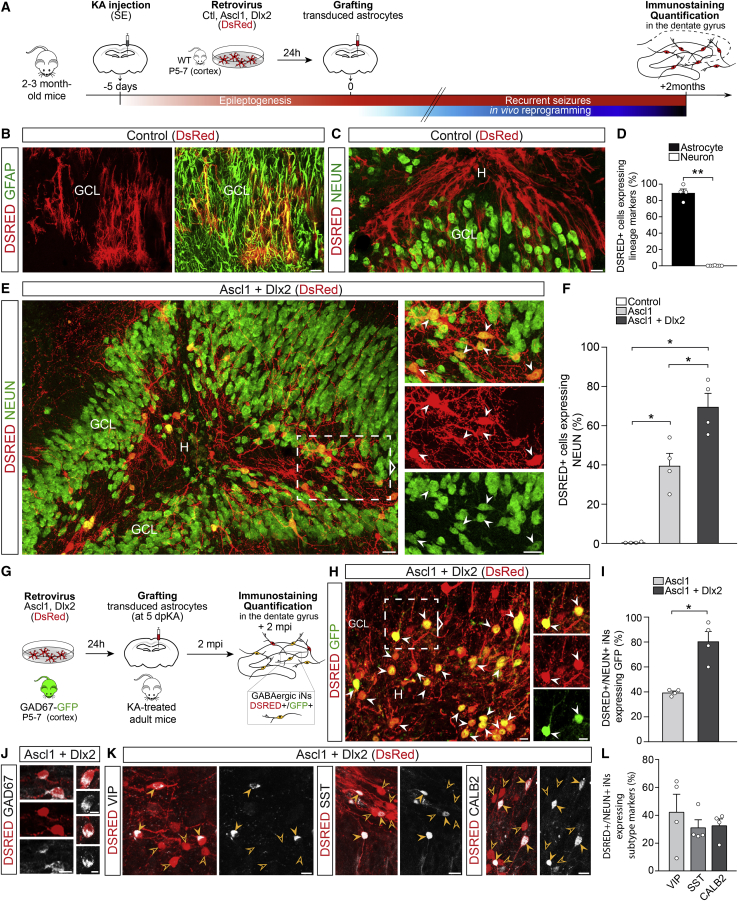
*In vivo* reprogramming of grafted cortical astroglia into GABAergic iNs within the MTLE-HS mouse hippocampus (A) Experimental procedures. (B and C) DSRED+ grafted astroglia transduced with the control retrovirus (DsRed) express GFAP, 2 mpi (B). None of the DSRED+ cells express NEUN (C). (D) Proportion of control-transduced astroglia (DSRED) expressing astrocytic (GFAP; n = 4) or neuronal markers (DCX, MAP2, or NEUN; n = 6), 2 mpi. (E) DSRED/NEUN+ iNs (arrowheads) derived from grafted astroglia transduced with the Ascl1/Dlx2-encoding retrovirus (DsRed), 2 mpi. Note the pronounced dispersion of dentate GCs induced by KA. (F) Proportion of DSRED+ cells converted into NEUN+ iNs following expression of Ascl1 (n = 4), Ascl1/Dlx2 (n = 4) or control (n = 4), 2 mpi. (G–I) Conversion of astroglia isolated from GAD67-GFP mice into GABAergic iNs. (G) Experimental procedures. (H) Ascl1/Dlx2-iNs (DSRED) expressing GFP (arrowheads; 2 mpi) demonstrating their GABAergic identity. (I) Proportion of DSRED/NEUN+ iNs expressing GFP following Ascl1 (n = 4) or Ascl1/Dlx2 (n = 4) reprogramming, 2 mpi. (J) Ascl1/Dlx2-iNs (DSRED) express GAD67. (K) Ascl1/Dlx2-iNs (DSRED) expressing VIP, SST, or CALB2 (full arrowheads). Empty arrowheads point to marker-negative iNs. (L) Proportion of DSRED/NEUN+ iNs expressing VIP, SST, or CALB2 (n = 4 each). Bars, mean ± SEM. Statistical analysis (D, F, and I): two-tailed Mann-Whitney test. ^∗^p < 0.05, ^∗∗^p < 0.01. Right panels (E and H): magnified views of boxed areas. Composite images, (C) and (E). Scale bars: 25 μm (B, C, and E), 10 μm (H, J, and K). GCL, granule cell layer; H, hilus. See also Figures S1 and S2.

We previously showed that retrovirally driven expression of the TFs Ascl1 and Dlx2 instructs reprogramming of postnatal cortical astroglia *in vitro* to generate functional, synapse-forming GABAergic iNs (Heinrich et al., 2010; 2011a). To explore whether *in vivo* glia-to-neuron conversion could be achieved within an epileptic hippocampus, we took advantage of the reprogramming competence of these cortical astroglia and examined whether they could also be converted *in vivo* into GABAergic iNs when grafted in the MTLE-HS mouse hippocampus. To this end, cortical astrocytes were transplanted in the dentate gyrus of the sclerotic hippocampus at 5 dpKA, directly after their *in vitro* transduction (24 h) with retroviruses encoding Ascl1 and Dlx2 (together with DsRed reporter) to induce their reprogramming *in vivo*, or encoding DsRed-only for control (Figure 1A).

Control-transduced astroglia remained committed to their lineage as revealed by astrocyte morphology and GFAP expression at 2 months post-infection (mpi; >90% of DSRED+ cells; Figures 1B, 1D, and S2A). Virtually none of the control cells expressed the neuronal markers DCX, NEUN, or MAP2 (1.5%; Figures 1C, 1D, and S2B), thus demonstrating the absence of spontaneous astroglia-to-neuron conversion within the epileptic hippocampus environment. In sharp contrast, the combined expression of Ascl1 and Dlx2 instructed astroglia to generate iNs with high efficiency. About 70% of Ascl1/Dlx2-transduced cells expressed the typical neuronal marker NEUN (Figures 1E and 1F) and ∼80% extended MAP2+ dendrites at 2 mpi (Figures S2E and S2F). Consistent with glia-to-neuron fate conversion, the acquisition of mature neuronal traits was progressive as illustrated by the initial expression of the immature neuronal marker DCX in iNs at 10 days post-infection (dpi; Figure S2C). At 2 mpi, NEUN+ iNs, whose somata were primarily located in the hilus and GC layer, exhibited complex neuronal morphologies indicative of advanced maturation and extended their dendrites and axons throughout the dentate gyrus of the dorsal hippocampus, thus creating dense fiber networks (Figures 1E and S2D). We observed that Ascl1-only expressing astroglia could also be redirected toward mature neurons *in vivo*, albeit with more moderate efficiency (Figures 1F and S2G).

We next asked whether iNs acquired a GABAergic identity and grafted astroglia isolated from GAD67-GFP mice (Figure 1G) from which astroglia-derived GABAergic iNs will turn on GFP under the GAD67 promoter. Strikingly, ∼80% of NEUN+ iNs generated upon forced Ascl1/Dlx2 co-expression differentiated into GABAergic neurons as revealed by GFP reporter co-expression at 2 mpi (Figures 1H and 1I), whereas merely a modest fraction of Ascl1-only iNs entered the GABA lineage (Figures 1I and S2H). Consistent with GFP reporter expression, NEUN+ iNs expressed the GAD67 protein (Figure 1J). Interestingly, a substantial fraction of NEUN+ iNs acquired expression of calretinin (CALB2; ∼30%), somatostatin (SST; ∼30%), or vasoactive intestinal peptide (VIP; ∼40%) (Figures 1K and 1L), thus suggesting some degree of specification toward distinct interneuron subtypes. Taken together, these results show that direct neuronal conversion of astroglia can be achieved with high efficiency within the epileptic environment in the adult MTLE-HS mouse hippocampus and that astroglia-derived iNs acquire a GABAergic identity.

### Retroviruses target reactive glial cells, but not neuronal-restricted progenitors, in the MTLE-HS hippocampus

**Figure 2:**
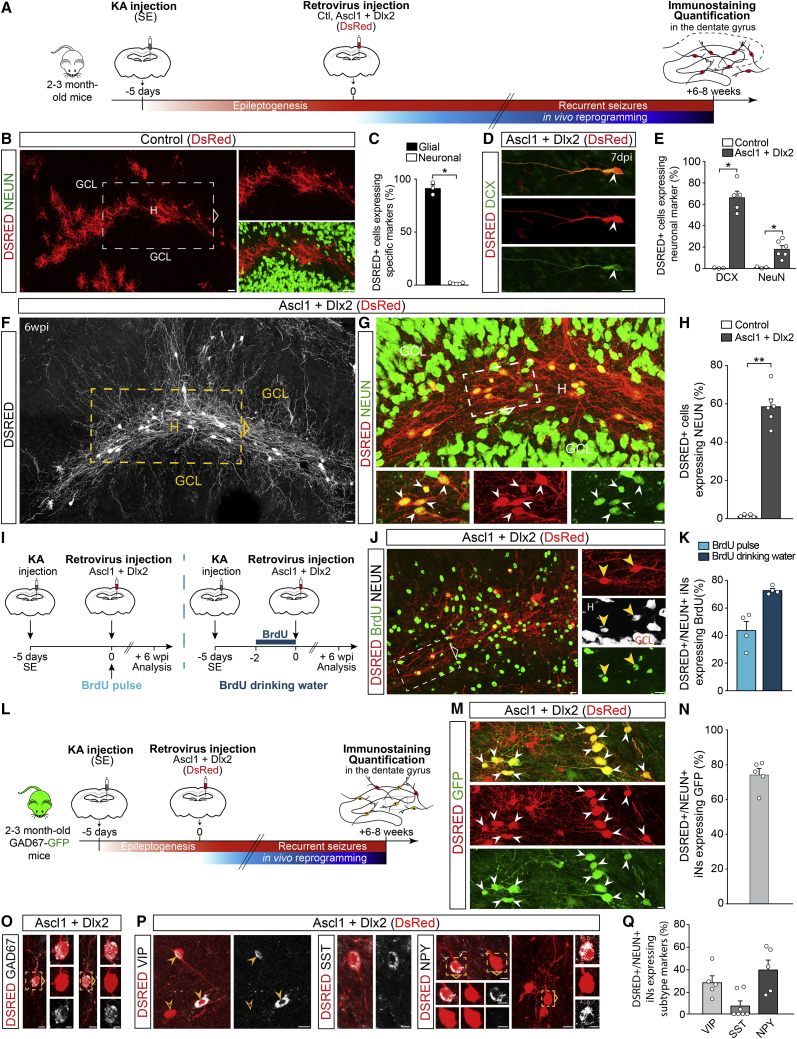
*In vivo* reprogramming of reactive hippocampal glia into GABAergic iNs in adult MTLE-HS mice (A) Experimental procedures. (B) DSRED+ hippocampal glia transduced with the control retrovirus (DsRed), 6 wpi. None of the DSRED+ cells express NEUN. (C) Proportion of control-transduced cells (DSRED) expressing glial (OLIG2, GFAP, or IBA1; n = 3) or neuronal marker (NEUN; n = 5), 6 wpi. (D–H) Hippocampal reactive glia transduced with the Ascl1/Dlx2-encoding retrovirus (DsRed) are reprogrammed into iNs. (D) DSRED+ iN expressing DCX, 7 dpi. (E) Proportion of DSRED+ cells expressing DCX or NEUN following expression of Ascl1/Dlx2 (DCX, n = 5; NEUN, n = 6) or control (n = 3), 7 dpi. (F) Ascl1/Dlx2-iNs (DSRED, white) exhibit complex neuronal morphologies and extend fibers creating dense networks throughout the dentate gyrus, 6 wpi. (G) Magnified views of the area boxed in (F) showing that Ascl1/Dlx2-iNs (DSRED) express NEUN (arrowheads), 6 wpi. (H) Proportion of DSRED+ cells converted into NEUN+ iNs following expression of Ascl1/Dlx2 (n = 6) or control (n = 5; same mice as in C), 6 wpi. (I–K) BrdU labeling of iNs. (I) Experimental procedures. (J) DSRED/NEUN+ iNs labeled by BrdU (arrowheads, drinking water protocol), 6 wpi. (K) Proportion of DSRED/NEUN+ iNs immunoreactive for BrdU following single BrdU pulse (n = 4) or BrdU supply in drinking water (n = 4), 6 wpi. (L–N) Conversion of hippocampal reactive glia into GABAergic iNs in GAD67-GFP mice. (L) Experimental procedures. (M) Ascl1/Dlx2-iNs (DSRED) expressing GFP (arrowheads; 6 wpi) demonstrating their GABAergic identity. (N) Proportion of DSRED/NEUN+ iNs expressing GFP following Ascl1/Dlx2 expression (n = 5), 6 wpi. (O) Ascl1/Dlx2-iNs (DSRED) express GAD67. (P) Ascl1/Dlx2-iNs (DSRED) expressing VIP, SST, or NPY (full arrowheads). Empty arrowhead points to a VIP-negative iN. (Q) Proportion of DSRED/NEUN+ iNs expressing VIP (n = 5), SST (n = 7), or NPY (n = 5), 8 wpi. Bars, mean ± SEM. Statistical analysis (C, E, and H): two-tailed Mann Whitney test. ^∗^p < 0.05, ^∗∗^p < 0.01. Right (B and J) and bottom (G) panels: magnified views of boxed areas. Composite images, (B), (F), and (G). Scale bars: 10 μm except 25 μm (B and F). See also Figure S3.

Given that the epileptic environment proved to be permissive to glia-to-interneuron reprogramming, we next aimed at developing a strategy of converting endogenous hippocampal glia into GABAergic iNs in MTLE-HS mice *in situ*. More specifically, we took advantage of the fact that MoMLV (Moloney murine leukemia virus)-based retroviruses stably transduce proliferative cells only (Roe et al., 1993), to target expression of neuronal conversion genes to reactive glia undergoing proliferation in response to KA-induced injury (Heinrich et al., 2006; Kralic et al., 2005). Importantly, we and others previously demonstrated the cessation of adult dentate neurogenesis in the MTLE-HS mouse hippocampus (Heinrich et al., 2006; Kralic et al., 2005; Sierra et al., 2015) resulting from the depletion of neural stem cells (Sierra et al., 2015). First, to confirm the complete loss of endogenous neurogenesis, we scrutinized any remaining germinal activity in the dentate gyrus by supplying bromodeoxyuridine (BrdU) in drinking water for 3, 5, or 7 consecutive days after KA injection or saline for control (Figure S3A). Contrary to what was observed in saline-treated mice, virtually all BrdU+ cells expressing DCX in the ipsilateral dentate gyrus had disappeared by 5 dpKA (Figures S3B and S3C), thus confirming an early arrest of dentate neurogenesis. Thus, we selected 5 dpKA as a suitable time point for retroviral injection to target reactive glia in the absence of ongoing physiological neurogenesis. Indeed, following injection of a control retrovirus (DsRed only), virtually none of the DSRED+ transduced cells expressed the neuronal markers DCX and/or NEUN (<2%, Figures 2B, 2C, and S3I) or exhibited GC morphology at 4 dpi or 6 weeks post-infection (wpi).

Next, to determine which cell types proliferate in the sclerotic hippocampus at this time point, mice received a short pulse of BrdU at 5 dpKA and were sacrificed 2 h later (Figure S3D). Our quantifications revealed that 47% of BrdU+ cells were immunoreactive for OLIG2, while 13% and 38% expressed GFAP or IBA1, respectively (Figures S3E-S3F). We interpret these data as indicating that most proliferating cells belong to the oligodendroglial lineage (i.e., OPCs also referred to as NG2 glia), but we cannot exclude that some of the OLIG2+ cells may also comprise reactive astrocytes (Chen et al., 2008). Again, virtually none of the BrdU+ cells expressed DCX (<2%). Next, to determine which of these dividing cells were transduced by a retrovirus, mice received the control retrovirus encoding DsRed only at 5 dpKA and were sacrificed at 4 dpi (Figure S3G). Consistent with our BrdU labeling data, the majority of transduced cells were immunoreactive for OLIG2 (71%), while 14% were GFAP+ reactive astrocytes and 13% were IBA1+ microglia (Figures S3H and S3I, left bars). The fact that the same populations of proliferating glial cells were targeted by the two methods (Figures S3F and S3I, left bars) was further validated by directly combining BrdU and retroviral injections (Figure S3J), which resulted in co-labeling of the various glial cell populations at a high rate (Figures S3K–S3M) and in ratios (Figure S3N) comparable to those of the individual labeling methods alone (Figures S3F and S3I, left bars). Importantly, lineage tracing of control-transduced cells and their progeny at longer survival time (6 wpi) revealed that DSRED+ glial cells remained in their glial lineage without giving rise to any labeled neuron (Figures 2B, 2C, and S3I, right bars). Altogether, these data demonstrate that a retrovirus only transduces proliferating reactive glia, devoid of any neurogenic potential, in the MTLE-HS hippocampus. The absence of aberrant labeling of endogenous neurons allowed us in the following experiments to explore unambiguously glia-to-neuron reprogramming in the absence of labeling artifacts.

### *In vivo* reprogramming of reactive hippocampal glia into GABAergic iNs in adult MTLE-HS mice

**Figure 3:**
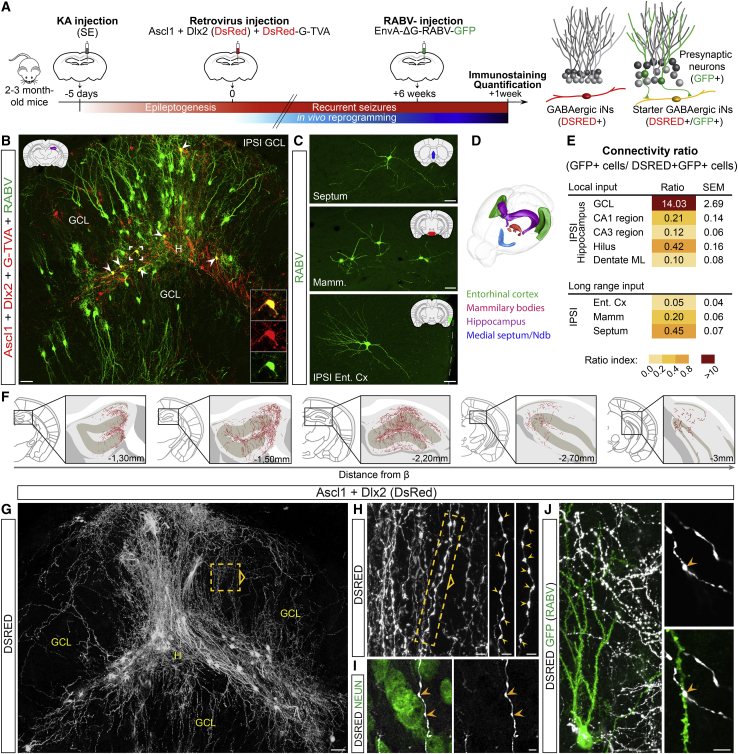
iNs derived from hippocampal reactive glia show widespread synaptic integration within the MTLE-HS mouse brain (A) Experimental procedures. (B–E) iNs receive synaptic innervation from endogenous neurons, 7 wpi. (B) DSRED/GFP+ starter iNs (arrowheads, insets) receive innervation from local GFP+ GCs. (C) Starter iNs receive innervation from GFP+ long-range projection neurons in the ipsilateral entorhinal cortex (Ent Cx), mammillary/supramammillary bodies (Mamm), and medial septum/nucleus of diagonal band (Ndb). Schematics in (B) and (C) highlight the brain structure shown in each panel. (D) 3D drawing shows brain regions establishing synapses onto iNs. (E) Numbers of GFP+ presynaptic neurons expressed as color-coded connectivity ratios, 7 wpi (mean ± SEM; n = 3). (F–J) iNs send axons impinging on GCs, 7 wpi (n = 8). (F) DSRED+ fibers from iNs extend over 1.7 mm along the rostro-caudal axis of the dorsal hippocampus. (G) Ascl1/Dlx2-iNs (DSRED, white) extend fibers forming dense networks throughout the dentate gyrus. (H) Magnified view of the area boxed in (G) shows iN axons and synaptic bouton-like structures (arrowheads). Right panels: magnified views of boxed area. (I) Synaptic boutons in close contact (arrowheads) with a NEUN+ GC soma. (J) iNs extend axons across dendritic arbors of GCs (GFP from RABV as in B). Synaptic boutons contact GC dendrites. Composite images, (B) and (G). Scale bars: 50 μm (B, C, and G), 5 μm (H–J). ML, molecular layer. See also Figure S4.

To reprogram reactive hippocampal glia into interneurons, MTLE-HS mice received hippocampal injection of a retrovirus encoding Ascl1 and Dlx2 (DsRed) at 5 dpKA (Figure 2A). In sharp contrast to control virus injection (Figures 2B and 2C), combined expression of Ascl1 and Dlx2 induced efficient reprogramming of hippocampal glia into iNs expressing the immature neuron marker DCX (67% of DSRED+ cells) and exhibiting an immature neuronal morphology at 7 dpi (Figures 2D and 2E), while few DSRED+ cells had already started to express NEUN (18%, Figure 2E). Consistent with progressive maturation, at longer survival time (6 wpi), iNs expressed NEUN (∼60% of DSRED+ cells; Figures 2G and 2H) and extended MAP2+ dendrites, with DCX being no longer detected. These iNs exhibited complex neuronal morphologies and extended several branched processes creating dense fiber networks throughout the MTLE-HS dentate gyrus (Figures 2F and 3G). Importantly, none of the iNs expressed PROX1 that is characteristic for dentate granule neurons (1.7% ± 0.1%). Next, to corroborate that *in situ* iNs were indeed generated *de novo*, we initially labeled dividing glial cells with BrdU prior to, or at the time of retrovirus injection (Figure 2I). Consistent with the restriction of retroviral transduction to cells undergoing division, these experiments revealed a high rate of BrdU and NEUN co-labeling in iNs at 6 wpi (Figures 2J and 2K).

To determine whether these iNs had acquired a GABAergic identity, we injected KA into the hippocampus of GAD67-GFP mice that subsequently received the retrovirus encoding Ascl1 and Dlx2 (DsRed) at 5 dpKA (Figure 2L). Notably, at 6 wpi, ∼75% of NEUN+ iNs had differentiated into GABAergic neurons as revealed by co-expression with GFP (Figures 2M and 2N). Furthermore, consistent with GFP expression, NEUN+ iNs expressed the GAD67 protein (Figure 2O). Finally, we found that a fraction of iNs expressed neuropeptide Y (NPY) or VIP (40% and 29%, respectively) and to a lesser extent SST (8%) at 8 wpi (Figures 2P and 2Q), thus revealing the acquisition of interneuron subtype-specific features. Taken together, our results provide strong evidence that proliferating hippocampal reactive glia are efficiently converted into newly generated GABAergic iNs in the adult MTLE-HS mouse hippocampus.

### Widespread synaptic integration of GABAergic iNs within the MTLE-HS mouse brain

The long-term survival and maturation of glia-derived iNs in the epileptic hippocampus prompted us to investigate their synaptic integration within endogenous networks in the adult MTLE-HS mouse brain by using rabies virus (RABV)-mediated retrograde monosynaptic tracing (Wickersham et al., 2007). To this end, resident hippocampal glia were co-transduced with a retrovirus encoding the reprogramming genes (Ascl1/Dlx2) and a retrovirus encoding both the RABV glycoprotein G and the TVA receptor for EnvA-pseudotyped RABV (Bergami et al., 2015; Deshpande et al., 2013). By 6 wpi, TVA-expressing iNs were selectively transduced with a GFP-encoding, EnvA-pseudotyped ΔG RABV that subsequently spread transsynaptically from these iNs (i.e., starter neurons) to their first-order presynaptic partners which then expressed GFP (Figure 3A).

When analyzed one week later (7 wpi), starter iNs received innervation from a large amount of local GFP+ presynaptic GCs, which were distributed throughout the entire dentate gyrus along the rostro-caudal axis of the dorsal hippocampus (Figure 3B). Per starter iN, we counted ∼14 GCs that were traced using the RABV (Figure 3E). A small number of GFP+ residual pyramidal cells was traced within the ipsilateral CA hippocampal subfields (Figure 3E) that was in agreement with extensive cell death in these areas (Figure S1C). Next, we examined whether iNs also received long-range connections from remote brain areas such as cortical and subcortical areas. Remarkably, we observed a moderate but consistent innervation by GFP+ long-range projection neurons located in the ipsilateral entorhinal cortex, the mammillary/supramammillary bodies, and the medial septum/nucleus of diagonal band of Broca (Figures 3C–3E). Connectivity ratios for these areas were, however, much lower than local innervation from GCs (Figure 3E).

We next investigated whether GABAergic iNs also sent efferent fibers to endogenous neurons and, in particular, to neighboring GCs that are the main neurons surviving in the MTLE-HS mouse hippocampus (Figure S1C) and likely represent the neuronal substrate for hippocampal seizure activity. By ∼2 mpi, iNs extended DSRED+ axonal fibers, as indicated by the presence of numerous synaptic bouton-like structures (Figure 3H), creating highly complex networks throughout the whole dispersed dentate gyrus (Figure 3G). Remarkably, DSRED+ fibers extended over ∼2 mm along the rostro-caudal axis of the dorsal hippocampus, thus innervating a considerable portion of the epileptic dentate gyrus (Figure 3F). We observed a massive amount of DSRED+ axons sneaking in between GCs (Figures 3G-3I) and axonal varicosities impinging on neighboring GC somata (Figure 3I). Combining RABV-monosynaptic tracing (GFP) with DSRED immunostaining, we found that iNs also extended axons across GFP+ dendritic arbors of GCs, forming *en passant* synaptic boutons on successive GC dendrites (Figure 3J). Together, these data suggest that iNs are both pre- and post-synaptic partners of GCs. Of note, iNs did not project their axons outside the MTLE-HS hippocampus, reminiscent of hippocampal GABAergic interneurons.

To study whether a similar degree of synaptic integration could be observed in the case of iNs derived from engrafted astroglia, the latter were transduced with both retroviruses (Ascl1/Dlx2 and G/TVA) prior to transplantation, and astroglia-derived iNs were subsequently transduced with EnvA-pseudotyped RABV at 2 mpi (Figure S4A). Remarkably, we observed a similar integration pattern with iNs receiving massive innervation by local GFP+ GCs (Figures S4B and S4E) alongside moderate inputs from GFP+ long-range projection neurons (Figures S4C–S4E). Moreover, iNs sent a considerable number of axonal fibers exhibiting numerous synaptic bouton-like structures in close contact with dentate GC somata and dendrites (Figures S4F–S4I), closely matching the widespread distribution pattern observed for iNs derived from hippocampal glia (Figures 3F–3J). Altogether, our results demonstrate that, after long-term survival, iNs not only receive local and long-range afferent connectivity but also extend axons putatively forming synapses with GCs.

### Glia-derived iNs are physiologically functional and establish GABAergic synapses with GCs

**Figure 4:**
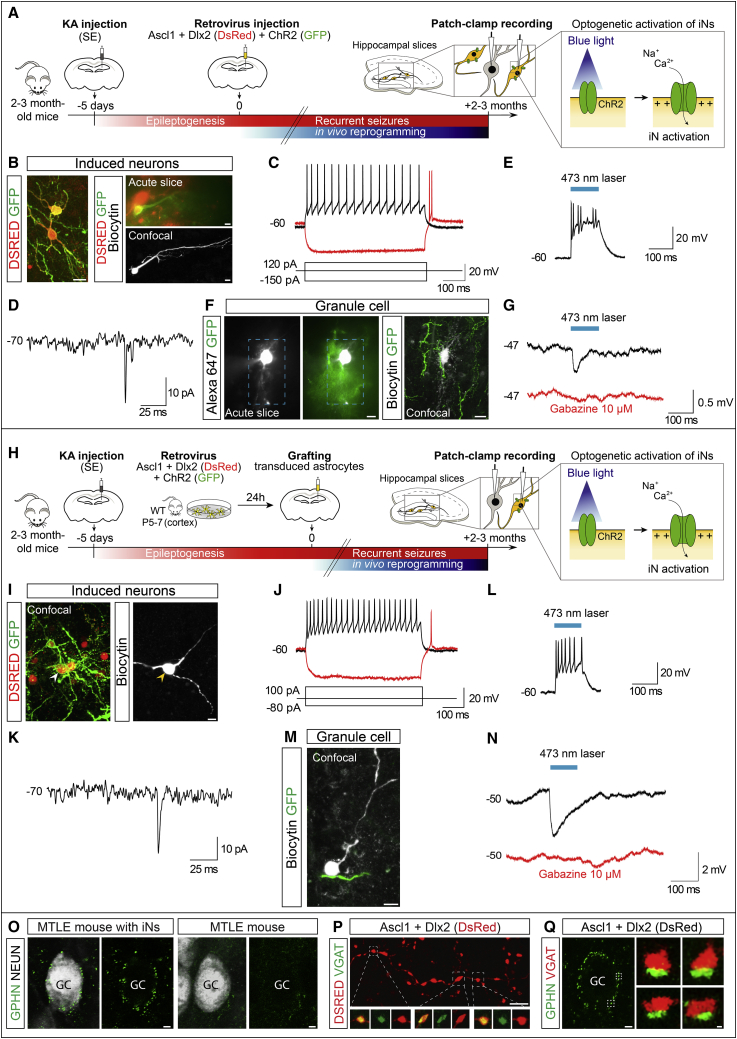
iNs are physiologically functional and form GABAergic synapses with GCs in the MTLE-HS hippocampus (A–G) iNs derived from hippocampal reactive glia. (A) Experimental procedures. (B) Left: Ascl1/Dlx2-iNs (DSRED) expressing CHR2 (GFP). Right: example of recorded DSRED/CHR2+ iN visualized in acute slice (top), and after recording and intracellular biocytin injection (bottom). (C) iN showing repetitive action potential firing in response to depolarizing current injection (black; 4 iNs recorded, n = 3 mice). Rebound spiking generated following relief from hyperpolarization (red; 3 of 4 iNs). (D) Spontaneous synaptic input recorded from an iN (3 of 3 iNs, n = 3 mice). (E) DSRED/CHR2+ iN showing action potential firing in response to blue light stimulation (473 nm, 100 ms; 4 of 4 iNs, n = 3 mice). (F) Example of recorded GC (filled with Alexa 647) surrounded by CHR2+ (GFP) iN processes visualized in acute slice (left), and after recording and intracellular biocytin injection (right). Blue box: area of laser stimulation. (G) GABAergic IPSPs recorded in a GC (average trace of 5 consecutive responses is shown) evoked by blue light stimulation of CHR2+ iNs (black; 4 of 8 GCs, n = 3 mice). IPSPs were blocked by gabazine (red; 4 of 4 GCs, n = 3 mice). (H–N) iNs derived from grafted cortical glia. (H) Experimental procedures. (I) Recorded DSRED/CHR2+ iN filled with biocytin (arrowhead). (J) Repetitive action potential firing (black; 4 iNs recorded, n = 4 mice) and rebound spike (red; 3 of 4 iNs) recorded in an iN as in (C). (K) iNs receive spontaneous synaptic input (3 of 4 iNs, n = 4 mice). (L) iN activation by blue light (4 of 4 iNs, n = 4 mice). (M) Recorded GC (filled with biocytin) surrounded by a CHR2+ iN process. (N) GABAergic IPSPs recorded in a GC as in (G) (black; 6 of 8 GCs, n = 5 mice) and blocked by gabazine (red, 4 of 4 GCs, n = 4 mice). (O–Q) Immunohistological evidence for GABAergic synapses between iNs and GCs. (O) Left: example of a GC (white) exhibiting gephyrin+ (GPHN) puncta outlining its soma in MTLE-HS mice after Ascl1/Dlx2-reprogramming, 2 mpi. Right: very few gephyrin+ puncta in MTLE-HS mice without iNs. (P) DSRED+ axon from a GABAergic iN showing axonal varicosities containing clusters of VGAT+ puncta. (Q) Gephyrin+ puncta around a GC soma (left) and examples of close apposition of gephyrin+ and VGAT+ puncta (right) in mice with GABAergic iNs. Scale bars: 10 μm (B, F, I, and M), 2 μm (O–Q). See also Figure S5.

Next, to assess whether iNs are physiologically functional and establish GABAergic synapses with GCs, we followed an optogenetic strategy based on selective expression of channelrhodopsin2 (ChR2) in iNs, thereby allowing their blue light-mediated activation. To this end, we simultaneously targeted retroviral expression of the reprogramming genes Ascl1/Dlx2 (DsRed) and ChR2 (GFP) to hippocampal reactive glia through direct retrovirus injection *in situ* or to cortical astroglia prior to grafting into the epileptic hippocampus (Figures 4A and 4H). By 2–3 mpi, acute hippocampal slices of MTLE-HS mice were prepared, and we performed whole-cell patch-clamp recordings of iNs (identified by fluorescent reporters) and GCs. First, we found that iNs in both reprogramming settings were capable of repetitive action potential firing in response to depolarizing step-current injection (Figures 4B, 4C, 4I, 4J, and S5A–S5E). Moreover, following the relief from prolonged hyperpolarization, most iNs generated a rebound spike reminiscent of low-threshold spiking interneurons (Figures 4C and 4J; Ibáñez-Sandoval et al., 2011; Kawaguchi, 1995). In addition, some iNs also displayed a time-dependent sag in response to hyperpolarizing current pulses due to an Ih-like current, another characteristic of low-threshold spiking interneurons (Figure S5F; Ibáñez-Sandoval et al., 2011; Kawaguchi, 1995). Together, these data demonstrate that iNs derived from hippocampal glia *in situ* or grafted astroglia acquire properties of physiologically functional neurons.

Next, to examine whether iNs form synaptic connections with endogenous GCs, CHR2+ iNs were stimulated by brief laser pulses while recording the membrane potential from GCs in current-clamp. CHR2+ iNs in both experimental paradigms (*in situ* reprogramming and grafting of transduced astroglia) were reliably activated by blue light eliciting action potential firing with short onset latency (Figures 4E and 4L). In both reprogramming paradigms, blue light-mediated stimulation of iNs consistently evoked inhibitory postsynaptic potentials (IPSPs) with a fast onset (3.5 ms) in GCs that were surrounded by GFP+ (hence CHR2+) iN processes (Figures 4F, 4G, 4M, 4N, and S5J). Importantly, IPSPs were blocked by gabazine (Figures 4G and 4N), a selective antagonist of GABA_A_ receptors located at the synaptic cleft (Wlodarczyk et al., 2013). In addition, synaptic potentials reverted around the calculated Cl^−^ reversal potential in our experimental conditions (−70 mV; Figures S5G and S5H). Furthermore, we could observe that blue light-mediated iN activation resulted in transient inhibition of action potential firing in GCs (Figure S5I). We also observed that iNs received spontaneous synaptic input from the surrounding network (Figures 4D and 4K), which is consistent with our results from RABV-mediated synaptic tracing and suggests that network activity can recruit iN-mediated inhibition of GCs. Consistent with these results, we found clusters of vesicular GABA transporter (VGAT)-positive puncta within the axonal varicosities along DSRED+ axons of iNs (Figure 4P). We also observed that GCs exhibited puncta immunoreactive for the GABA_A_ receptor scaffold protein gephyrin, outlining their somata in hippocampi containing GABAergic iNs (Figure 4O, left), whereas very few gephyrin+ puncta were observed in animals without iNs (control virus, Figure 4O, right). Finally, we could observe close appositions of gephyrin+ and VGAT+ puncta in mice with iNs (Figure 4Q). Taken together, these data provide strong evidence that iNs establish functional GABAergic synapses onto GCs.

### *In vivo* reprogramming of glial cells into GABAergic iNs reduces spontaneous recurrent hippocampal seizures in MTLE-HS mice

**Figure 5:**
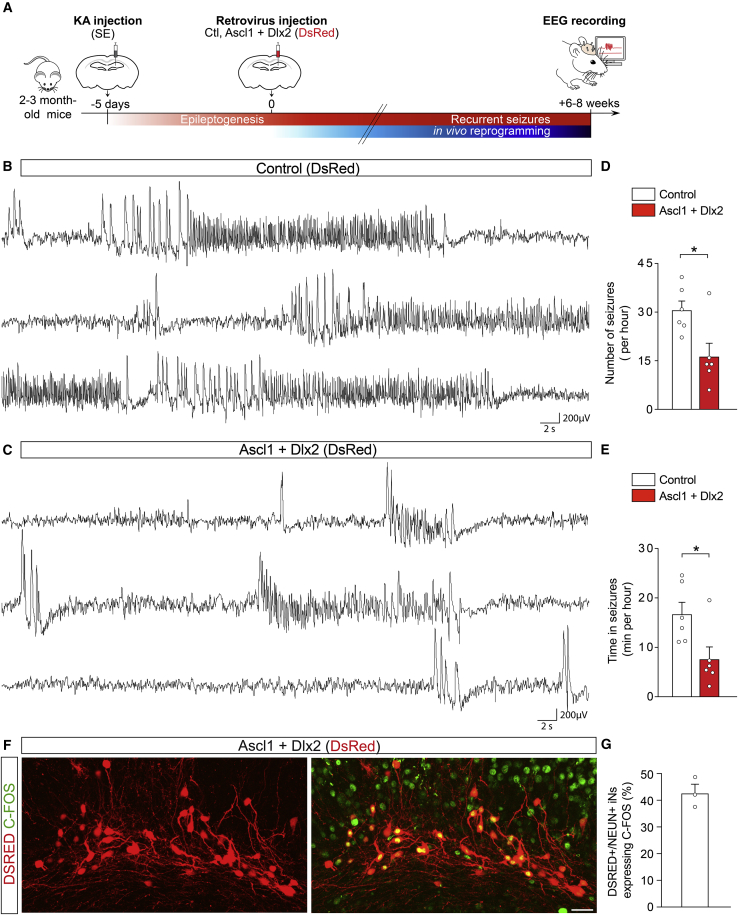
*In vivo* reprogramming of hippocampal reactive glia into GABAergic iNs reduces spontaneous recurrent hippocampal seizures in MTLE-HS mice (A) Experimental procedures. (B) Intrahippocampal EEG recording from a MTLE-HS mouse injected with the control retrovirus (DsRed) show numerous non-convulsive EEG seizures consisting of slow rhythmic high-voltage sharp waves followed by higher-frequency and lower-amplitude spikes (6–8 wpi). (C) Intrahippocampal EEG recording from a MTLE-HS mouse injected with the Ascl1/Dlx2-retrovirus show drastic decrease in number of hippocampal EEG seizures and time spent in seizures (6–8 wpi). (D and E) Number of hippocampal EEG seizures (D) and time spent in seizures (E, cumulative seizure duration, min/h) in MTLE-HS mice injected with the Ascl1/Dlx2-retrovirus (n = 6) or control retrovirus (n = 6), 6–8 wpi. Statistical analysis: two-tailed Mann-Whitney test. ^∗^p < 0.05. (F and G) A substantial fraction of iNs is physiologically active. (F) Numerous Ascl1/Dlx2-iNs (DSRED) express C-FOS, 8 wpi. (G) Proportion of DSRED/NEUN+ iNs immunoreactive for C-FOS, 6–8 wpi (n = 3). Representative traces in (B) and (C) show EEG recordings (3 min each) in the KA-injected hippocampus. Bars, mean ± SEM. Scale bar: 25 μm. See also Figure S6.

The specification of iNs into GABAergic neurons and their ability to form GABAergic synapses onto GCs prompted us to investigate whether iNs derived from hippocampal reactive glia reduce the recurrence of spontaneous hippocampal seizures during the chronic phase of the disease. To this end, we recorded electroencephalographic (EEG) activity in the KA-injected hippocampus 6–8 weeks after injection of the retrovirus encoding Ascl1/Dlx2 or the control retrovirus (Figure 5A), i.e., several weeks after epilepsy development was completed in all mice and had led to the occurrence of recurrent seizures, which is in agreement with our previous studies showing chronic epilepsy in all KA-injected mice in this model (Heinrich et al., 2011b; Riban et al., 2002). Given that MTLE-HS mice develop focal, non-convulsive electrographic seizures with rare secondary generalization (Löscher et al., 2020) similarly to seizures described in human MTLE-HS (Cendes et al., 2014), we quantified the number and duration of hippocampal EEG seizures in line with classical seizure analysis in this model (see STAR Methods).

Following injection of the control retrovirus, spontaneous recurrent EEG seizures displayed the typical seizure pattern mostly consisting of slow rhythmic high-voltage sharp waves followed by higher-frequency and lower-amplitude spikes (Figure 5B), as previously described (Arabadzisz et al., 2005; Heinrich et al., 2011b; Maroso et al., 2010; Riban et al., 2002). Control virus-injected MTLE-HS mice displayed an average number of seizures (∼30 seizures/h) and cumulative seizure duration (i.e., time spent in seizures, ∼17 min/h; Figures 5D and 5E, white bars) similar to what has been described in this model (Arabadzisz et al., 2005; Duveau et al., 2016; Maroso et al., 2010; Stamboulian-Platel et al., 2016). In sharp contrast, following injection of the Ascl1/Dlx2-encoding retrovirus, the number of spontaneous recurrent seizures was significantly decreased compared to control animals (i.e., suppression of ∼15 seizures/h; Figures 5C and 5D, red bars). Ascl1/Dlx2-injected mice also spent drastically less time in seizure state compared to controls (Figure 5E, red bars). These data indicate that GABAergic iNs derived from Ascl1/Dlx2-reprogrammed hippocampal glia have the ability to reduce the recurrence of spontaneous seizures during the chronic phase of the disease. Consistent with seizure reduction mediated by Ascl1/Dlx2-iNs, we observed at the end of the experiments dense networks of iNs within the dispersed dentate gyrus in all Ascl1/Dlx2 virus-injected mice recorded (Figure S6A). Importantly, histological analysis also revealed a similar extent of neuron loss in the CA subfields of Ascl1/Dlx2- and control-injected MTLE-HS mice (Figures S6A–S6C), confirming a similar degree of KA-induced hippocampal sclerosis in all animals. Altogether, these data demonstrate that *in vivo* reprogramming of resident hippocampal glia into GABAergic iNs significantly decreases chronic seizure activity in MTLE-HS mice.

**Figure 6:**
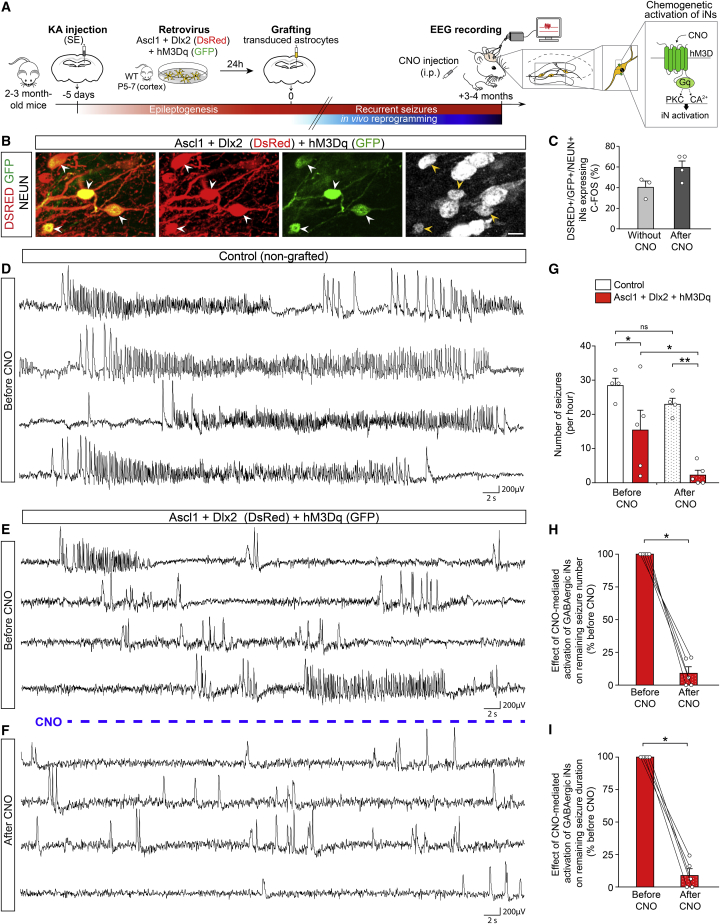
Chemogenetically enhancing iN activity results in complete seizure suppression in MTLE-HS mice (A) Experimental procedures. (B) NEUN+ (white) iNs derived from grafted astroglia expressing Ascl1/Dlx2 (DSRED) and hM3Dq (GFP, arrowheads), 4 mpi. (C) Proportion of DSRED/NEUN/GFP+ iNs immunoreactive for C-FOS in absence of CNO (n = 3) and after CNO treatment (n = 4), 3–4 mpi. (D) Intrahippocampal EEG recording from a control non-grafted MTLE-HS mouse showing recurrence of numerous EEG seizures. (E and F) Intrahippocampal EEG recordings from a MTLE-HS mouse with hM3Dq+ GABAergic iNs before (E) and after CNO treatment (F), 3–4 mpi. (E) Drastic decrease in the number of EEG seizures in absence of CNO compared to control non-grafted MTLE-HS mice (D). (F) CNO-evoked activation of hM3Dq+ iNs suppresses remaining EEG seizure activity below reducing effects of iNs in absence of CNO (E). Only residual isolated spikes remain visible. (G) Number of hippocampal EEG seizures in MTLE-HS mice with hM3Dq+ GABAergic iNs (red bars; n = 5) and control non-grafted MTLE-HS mice (white bars; n = 4) in absence of CNO (left bars) and after CNO treatment (right dotted bars). Same mice analyzed before and after CNO. Statistical analysis: repeated-measures two-way ANOVA followed by Sidak’s multiple comparison *post hoc* test. ^∗^p < 0.05, ^∗∗^p < 0.01. (H and I) Pairwise comparisons of seizure numbers (H) and time in seizures (I) before and after CNO treatment in each MTLE-HS mouse with hM3Dq+ GABAergic iNs (n = 5; same mice as in G red bars). Statistical analysis: one-tailed Wilcoxon matched-pairs test. ^∗^p < 0.05. Lines connect data from individual mice before and after CNO. Representative traces in (D)–(F) show EEG recordings (4 min each) in the KA-injected hippocampus. Bars, mean ± SEM. Scale bar: 10 μm. See also Figure S6.

Given that iNs derived from engrafted astroglia also establish GABAergic synapses onto GCs, we next examined whether they could also reduce chronic seizure activity, while also assessing the durability of such effect (Figure 6A). Toward this end, we recorded hippocampal EEG activity in MTLE-HS mice 3-4 months after grafting of Ascl1/Dlx2-transduced glia. Remarkably, Ascl1/Dlx2-iNs led to a significant reduction in the number of hippocampal recurrent seizures compared to control non-grafted MTLE-HS mice (Figures 6D, 6E, and 6G, left bars), and this reduction was comparable to the one observed with iNs derived from resident hippocampal glia (Figure 5D). Consistent with long-term seizure reduction, immunostaining in these EEG-recorded mice evidenced long-term survival of graft-derived iNs forming dense networks (up to 7 mpi, Figure S6D). Again, a similar extent of KA-induced neuronal loss was also observed in the CA subfields in all mice (data not shown). Taken together, these grafting data provide additional evidence for the ability of GABAergic iNs to reduce chronic seizure activity in MTLE-HS mice.

### Chemogenetically enhancing iN activity results in complete seizure suppression in MTLE-HS mice

To assess which proportion of iNs (*in situ* iNs or graft-derived iNs) were physiologically active during the chronic phase of the disease characterized by recurrent seizures, we took advantage of the transient induction of the immediate early gene c-Fos as a readout for highly active neurons (Yassin et al., 2010). In both reprogramming paradigms, immunostaining revealed that ∼40% of NEUN+ iNs exhibited C-FOS+ nuclei (Figures 5F, 5G, and 6C, left bar). These data therefore indicate that a substantial fraction of iNs is active at a suprathreshold level for C-FOS detection, whereas the activity of the other iNs remains below detection threshold. Thus, we hypothesized that the overall level of iN activity had not reached its ceiling and therefore could be further increased to enhance the seizure-suppressing effect of iNs. To test this possibility, we followed a chemogenetic approach based on specific expression of excitatory DREADDs (designer receptor exclusively activated by designer drugs) (Urban and Roth, 2015) in GABAergic iNs, thereby allowing for their selective activation. To this end, we took advantage of the fact that prior to grafting, astroglia could be efficiently engineered *in vitro* to express excitatory DREADDs alongside reprogramming TFs. Astroglia were co-transduced with a retrovirus encoding the excitatory Gq-coupled mutated human M3 muscarinic receptor (hM3D_q_-GFP) and a retrovirus encoding Ascl1/Dlx2 (DsRed), 24 h before being grafted into the dentate gyrus of MTLE-HS mice and being allowed for reprogramming *in vivo* (Figure 6A). By 3–4 mpi, we monitored intrahippocampal EEG activity first in the absence and then in the presence of the hM3D_q_ ligand, clozapine-N-oxide (CNO) in the same mice, to examine the effect of selectively enhancing activity in hM3D_q_+ iNs (NEUN+) (Figures 6A and 6B). For control, EEGs were also acquired in non-grafted MTLE-HS mice before and after CNO treatment.

We first showed in control non-grafted MTLE-HS mice that CNO administration on its own had no significant effect on the number and cumulative duration of recurrent seizure activity (Figures S6E–S6G and 6G, white bars), which is in agreement with previous reports in various epilepsy models (Cǎlin et al., 2018; Kätzel et al., 2014; Zhou et al., 2019). In sharp contrast, CNO treatment of animals harboring hM3D_q_+ iNs almost entirely suppressed, in each mouse, the remaining EEG seizure activity below the reducing effects of iNs in absence of CNO, as reflected by a dramatic reduction in the number of seizures after CNO compared with recordings before CNO (Figures 6E, 6F, and 6G, red bars). Pairwise comparisons in mice with hM3D_q_+ iNs further evidenced that CNO-evoked recruitment of iNs produced a ∼90% reduction in both the number and cumulative duration of residual seizures compared to recordings before CNO (Figures 6H and 6I). In fact, only residual low-frequency and isolated EEG spikes remained visible after CNO treatment (Figure 6F). Consistent with more pronounced effects of hM3D_q_+ iNs on seizure activity following CNO treatment, we observed an increase in the proportion of C-FOS+ iNs after CNO (60%; Figure 6C). Taken together, these data strongly suggest that the level of iN activity is crucial for their therapeutic effect on epileptic activity.

## Discussion

In the present study, we show that both endogenous and grafted glia can be reprogrammed into GABAergic iNs in a mouse model of chronic MTLE-HS by forced expression of Ascl1 and Dlx2. Moreover, these iNs are functional and form GABAergic synapses with GCs. Finally, we show that GABAergic iNs promote a significant reduction in spontaneous recurrent seizure activity during the chronic phase of the disease. Collectively, our findings uncover *in vivo* glia-to-neuron reprogramming as a potential cell-based strategy to combat seizures in drug-resistant epilepsy.

We confirmed that intrahippocampal KA injection triggers an extensive loss of interneurons across all subtypes in the hippocampus, while at the same time abrogating endogenous adult dentate neurogenesis (Heinrich et al., 2006; Kralic et al., 2005; Sierra et al., 2015). In response to this KA-induced injury, we observed massive proliferation of reactive glial cells. Our strategy aimed at targeting these dividing glia for reprogramming into interneurons. We provide several lines of evidence for genuine reprogramming of dividing glia and *de novo* induced neurogenesis. First, we demonstrated that—in the absence of ongoing adult dentate neurogenesis—our retroviral vectors exclusively transduced dividing reactive glia. Second, iNs were efficiently labeled by BrdU, which was initially incorporated into proliferative glia prior to or around the time of retrovirus injection inducing reprogramming. Third, iNs transiently expressed DCX, indicating that reprogramming involved a transition through immature neuronal stages similar to physiological neurogenesis. Together, these data provide compelling evidence that our retrovirus-based approach evades aberrant labeling of endogenous neurons (Wang et al., 2021).

Following two reprogramming paradigms, our study reveals that hippocampal glia, but also cortical glia grafted in the epileptic hippocampus, can be converted into GABAergic iNs by forced expression of Ascl1 and Dlx2, which are known to play a major role in differentiation and subtype specification of interneurons during development (Lim et al., 2018; Lindtner et al., 2019). Interestingly, a substantial fraction of iNs from both origins expressed interneuron subtype-specific markers (e.g., VIP, SST, CALB2, or NPY) and exhibited firing patterns reminiscent of low-threshold spiking interneurons (Ibáñez-Sandoval et al., 2011; Kawaguchi, 1995). In contrast, we did not detect parvalbumin expression or fast spiking action potential firing. The induction of interneurons described here is unlikely to represent a complete restoration of the full spectrum of endogenous interneurons that will require further tuning of the reprogramming strategy (Dehorter et al., 2017). Moreover, future analysis will reveal to which extent iNs fully or only partially match known classes of interneurons both in terms of molecular identity and functional properties. Although iNs derived from glial cells of both hippocampal or neocortical provenance share many features, it remains to be examined to which extent differences in regional origin of the reprogrammed glia could result in region-specific functional properties (Herrero-Navarro et al., 2021). The fact that astroglia of dorsal telencephalic origin can give rise to interneurons, a neuron class that has its physiological origin in the ventral telencephalon, indicates that TFs can induce transcriptional programs that superimpose a new identity beyond the regional origin of the reprogrammed glia. As in the case of *in situ* direct reprogramming we likely also targeted NG2 glia, which may comprise cells of both dorsal and ventral telencephalic origin (Dimou and Gallo, 2015), it will be interesting to learn whether those of ventral origin exhibit greater competence toward adopting an interneuron fate in line with partially having shared similar patterning influences during development. In addition, extrinsic factors from the injured microenvironment may also have an impact on the reprogramming outcome (Heinrich et al., 2014) and promote iN survival, maturation, and subtype specification, as well as synaptic integration of the newly added neurons (Falkner et al., 2016; Gaillard et al., 2007).

We found clear evidence for functional network integration of iNs several weeks after their generation within the sclerotic hippocampus. RABV-synaptic tracing revealed that iNs received synaptic innervation not only from local GCs but also from long-range projection neurons located in remote brain areas. However, the most significant innervation stems from local GCs which undergo extensive rewiring of their axons (i.e., mossy fiber sprouting) in the MTLE-HS mouse hippocampus (Suzuki et al., 1997). In turn, the GC-mediated excitatory input is likely to promote survival, functional maturation, and subtype specification of GABAergic iNs, which is in line with activity-dependent mechanisms of survival and functional maturation of cortical interneurons during development (De Marco García et al., 2011; Wong et al., 2018). Conversely, GABAergic iNs give rise to bouton-like structures positive for presynaptic markers that impinge onto GCs that are one of the likely substrates of hippocampal seizure activity (Pun et al., 2012) and, hence, represent befitting target cells for iN-mediated inhibition to control seizures. Following an optogenetic approach, we obtained compelling evidence for GABAergic synaptic transmission between iNs (generated in both reprogramming paradigms) and GCs. This, together with the fact that iNs receive synaptic input from endogenous neurons, indicates that network activity can recruit inhibition of GCs mediated by iNs. Consistent with restoration, at least in part, of lost inhibitory synapses onto GCs, our study shows that GABAergic iNs have the capacity to reduce the recurrence of spontaneous hippocampal seizures during the chronic phase of the disease. Altogether, these data provide strong support for the notion that iNs promote seizure reduction through direct GABA-mediated synaptic inhibition. The fact that two independent strategies to provide GABAergic iNs yielded a similar reduction of seizure activity strongly argues for iNs being the main substrates for the antiepileptic effects. This is further corroborated by our observation that chemogenetically enhancing iN activity resulted in increased therapeutic impact on seizures. Besides providing effective GABA-mediated inhibition of GCs, an intriguing possibility is that the long-term presence of iNs could also induce seizure reduction by promoting a structural and functional rewiring of epileptic networks toward a generally less epileptic state.

There is a crucial need for effective therapeutic strategies to control seizures in MTLE-HS patients. The fact that GABAergic iNs significantly reduce chronic epileptic activity in MTLE-HS mice showing poor responsiveness to various antiepileptic drugs (Löscher et al., 2020) uncovers *in vivo* lineage reprogramming as a potential cell-based strategy to combat intractable MTLE-HS and possibly also other devastating forms of epilepsy. Importantly, although most hippocampal interneurons acutely succumb to excitotoxicity mediated by KA and an excess of glutamate release during the initial SE (Figures S1E and S1F; Bouilleret et al., 2000), regenerated GABAergic iNs showed long-term survival and integration within endogenous networks consistent with their long-term seizure-reducing effects. Whether discrete iN degeneration resulting from the recurrence of seizures may occur over time remains to be investigated. Finally, the fact that additional CNO-evoked activation of GABAergic iNs resulted in almost complete seizure suppression suggests that pharmacological activation of iNs could be used to boost their antiepileptic effects for translational perspectives.

### Limitations of study

Which subtypes of dividing hippocampal reactive glia (astrocytes, NG2 glia, and/or microglia) were converted into iNs and whether a differential cellular origin affected the reprogramming outcome remains to be determined. It is currently unknown whether reprogramming of reactive glia may result in remodeling of the microenvironment with potentially either beneficial or detrimental consequences for tissue homeostasis (Escartin et al., 2021; Liddelow et al., 2020). It also needs to be examined whether glial cell populations, despite reprogramming, are maintained through homeostatic control of glial cell proliferation (Hughes et al., 2013). Finally, regarding future clinical translation, non-invasive delivery methods of reprogramming genes may need to be envisaged such as systemic delivery of intravenously injected adeno-associated viruses, modified RNAs, or electromagnetized gold nanoparticles (Vignoles et al., 2019).

## STAR★Methods

### Key resources table

**Table:** 

REAGENT or RESOURCE	SOURCE	IDENTIFIER
**Antibodies**		

Rat monoclonal anti-BrdU (clone BU1/75 (ICR1), lot #GR191322-1) – dilution 1:200	Abcam	Cat#AB6326; RRID: AB_305426
Rat monoclonal anti-c-Fos (clone 108B5H5) – dilution 1:1000	Synaptic Systems	Cat#226 017 ; RRID: AB_2864765
Mouse monoclonal anti-Calretinin (clone 6B8.2, lot #2982300) – dilution 1:1000 (IHC), 1:500 (ICC)	Millipore	Cat#MAB1568; RRID: AB_94259
Guinea pig polyclonal anti-Doublecortin (lot #3059069) – dilution 1:200	Millipore	Cat#AB2253; RRID: AB_1586992
Chicken polyclonal anti-Green Fluorescent Protein (lot #GR3190550-14) – dilution 1:200 (IHC), 1:500 (ICC)	Abcam	Cat#ab13970; RRID: AB_300798
Mouse monoclonal anti-GFAP (clone 131-17719, lot #1819891) – dilution 1:200	ThermoFisher Scientific	Cat#A21282; RRID: AB_2535827
Mouse monoclonal anti-GAD67 (clone K-87) – dilution 1:500	Abcam	Cat#ab26116; RRID: AB_448990
Rabbit monoclonal anti-Gephyrin (clone RbmAb7a) – dilution 1:500	Synaptic Systems	Cat#147018; RRID: AB_2651176
Rabbit polyclonal anti-GFAP – dilution 1:500	Agilent (Dakocytomation)	Cat#Z0334; RRID: AB_10013382
Rabbit polyclonal anti-Iba1 (lot #LKN4881) – dilution 1:200	Wako Chemicals	Cat#019-19741; RRID: AB_839504
Chicken polyclonal anti-MAP2 – dilution 1:500	Synaptic Systems	Cat#188006; RRID: AB_2619881
Goat polyclonal anti-mCherry (lot #0081260214) – dilution 1:200 (IHC), 1:500 (ICC)	Sicgen	Cat#AB0081-200; RRID: AB_2333094
Guinea pig polyclonal anti-NeuN (lot #266004/2-16) – dilution 1:500	Synaptic Systems	Cat#266 004; RRID: AB_2619988
Rabbit polyclonal anti-NeuN (lot #3233110) – dilution 1:200	Millipore	Cat#ABN78; RRID: AB_10807945
Guinea pig polyclonal anti-Neuropeptide Y (lot #394004/1-1) – dilution 1:500	Synaptic Systems	Cat#394 004; RRID: AB_2721083
Guinea pig polyclonal anti-Parvalbumin – dilution 1/1000	Synaptic Systems	Cat# 195004; RRID: AB_2156476
Rabbit polyclonal anti-Olig2 (lot # 2276294) – dilution 1:200	Millipore	Cat# AB9610; RRID: AB_570666
Rabbit polyclonal anti Somatostatin-14 (lot#A18197) - dilution 1:500	Peninsula Laboratories	Cat#T-4103; RRID: AB_518614
Rabbit polyclonal anti-Red Fluorescent Protein (lot # 39707) – dilution 1:500	Rockland	Cat# 600-401-379; RRID: AB_2209751
Guinea pig polyclonal anti-vesicular GABA transporter (lot # 131003:37) – dilution 1:500	Synaptic Systems	Cat#131 003; RRID: AB_887869
Rabbit polyclonal anti-Vasoactive Intestinal Peptide (lot#1744002) – Dilution 1:500	Immunostar	Cat#20077; RRID: AB_572270

**Bacterial and virus strains**		

Retrovirus: RV-pCAG-Ascl1-IRES-DsRed	SBRI viral vector facility	N/A
Retrovirus: RV-pCAG-Dlx2-IRES-DsRed	SBRI viral vector facility	N/A
Retrovirus: RV-pCAG-Ascl1-p2A-Dlx2-IRES-DsRed	SBRI viral vector facility	N/A
Retrovirus: RV-pCAG-IRES-DsRed	SBRI viral vector facility	N/A
Retrovirus: RV-pCAG-DsRedExpress2-2A-G-IRES2-TVA	SBRI viral vector facility	N/A
Retrovirus: RV-pCAG-EGFP-2A-hM3Dq	SBRI viral vector facility	N/A
Retrovirus: RV-pUbi-ChR2-EGFP	SBRI viral vector facility	N/A
Pseudotyped Rabies Virus (RabV): SADΔG-EGFP(EnvA)	Laboratory of Karl-Klaus Conzelmann	N/A
One Shot TOP10 Chemically Competent *E. coli*	FisherScientific	Cat#10666493

**Chemicals, peptides, and recombinant proteins**		

Kainic acid monohydrate (Kainate)	Sigma-Aldrich	Cat#K0250; CAS: 58002-62-3
5-Bromo-2′-deoxyuridine (BrdU)	Sigma-Aldrich	Cat#B5002-5G; CAS: 59-14-3
SR 95531 hydrobromide (Gabazine)	Tocris	Cat#1262; CAS: 104104-50-9
Clozapine-N-oxide (CNO)	Enzo Life Sciences	Cat#BML-NS105-0005; CAS: 34233-69-7

**Experimental models: Cell lines**		

Retrovirus packaging cell line: 293GPG cells	Ory et al., 1996	N/A

**Experimental models: Organisms/strains**		

Mouse: C57BL/6J	Charles River	RRID:IMSR_JAX:000664
Mouse: heterozygous Gad1^tm1Tama^ (also called GAD67-GFP)	Laboratory of Takeshi Kaneko (Tamamaki et al., 2003)	MGI: 3590300

**Recombinant DNA**		

Plasmid: RV-pCAG-Ascl1-IRES-DsRed	Laboratory of Magdalena Götz (Heinrich et al., 2011a)	N/A
Plasmid: RV-pCAG-Dlx2-IRES-DsRed	Laboratory of Magdalena Götz (Heinrich et al., 2011a)	N/A
Plasmid: RV-pCAG-Ascl1-p2A-Dlx2-IRES-DsRed	This manuscript	N/A
Plasmid: RV-pCAG-IRES-DsRed	Laboratory of Magdalena Götz (Heinrich et al., 2011a)	N/A
Plasmid: RV-pCAG-DsRedExpress2-2A-G-IRES2-TVA	Laboratory of Magdalena Götz (Deshpande et al., 2013)	N/A
Plasmid: RV-pCAG-EGFP-2A-hM3Dq	Laboratory of Alejandro Schinder (Alvarez et al., 2016)	N/A
Plasmid: RV-pUbi-ChR2-EGFP	Laboratory of Shaoyu Ge (Groisman et al., 2020)	N/A
Plasmid: SADΔG-EGFP (EnvA)	Laboratory of Karl-Klaus Conzelmann (Wickersham et al., 2007)	N/A

**Software and algorithms**		

Coherence	Natus Deltamed	N/A
LAS AF	Leica	N/A
Zen 2.3	Zeiss	RRID: SCR_018163
ImageJ 1.51v	NIH	https://imagej.nih.gov/ij/; RRID: SCR_003070
Prism v.8	GraphPad	RRID: SCR_002798
Neurolucida 360	MBF Bioscience	RRID: SCR_016788
pClamp 10.7 software	Molecular Devices	N/A

**Other**		

LSM 710 confocal microscope	Zeiss	RRID: SCR_018063
LSM 880 confocal microscope	Zeiss	N/A
Airyscan module	Zeiss	N/A
TCS SPE confocal microscope	Leica	RRID: SCR_002140
TCS SP5 confocal microscope	Leica	RRID: SCR_018714
Slice Scope Pro 6000 System	Scientifica	N/A

### Resource availability

#### Lead contact

Further information and requests for resources and reagents should be directed to and will be fulfilled by the Lead Contact, Christophe Heinrich (christophe.heinrich@inserm.fr)

#### Materials availability

Unique resources and reagents generated in this study are available from the Lead Contact with a completed Material Transfer Agreement.

### Experimental model and subject details

#### Animals

Experiments were conducted on adult (2-3 months of age; 25-30 g) wild-type C57BL/6J male mice (Charles River, France; RRID: IMSR_JAX:000664) or Gad1^tm1Tama^ heterozygous male mice (also called GAD67-GFP, glutamic acid decarboxylase-green fluorescence protein; MGI: 3590300) (Tamamaki et al., 2003). For breeding, heterozygous GAD67-GFP mice were backcrossed to C57BL/6J mice. Mice were housed in Plexiglas cages (group-housed) with food and water *ad libitum* and kept in 12:12h light/dark cycles (room temperature = 22 ± 1°C). All animal procedures were carried out in accordance with the guidelines from the European Union directive (2010/63/EU) and approved by the French Ministry of Higher Education, Research and Innovation (APAFIS #12199-2017111523452655 and #12168-2017111219556152). All efforts were made to avoid animal suffering and to reduce animal numbers. Mice were randomly assigned to experimental groups.

#### Primary cell cultures

Primary cultures of astroglia from the postnatal mouse cerebral cortex were prepared and maintained as previously described (Heinrich et al., 2010, 2011a). Astroglia were isolated from the cerebral cortex of postnatal wild-type C57BL/6J mice or heterozygous GAD67-GFP mice at the age of postnatal day 5–7 (P5–P7; At that age sex could not be determined). Astroglial cells were cultured in uncoated plastic flasks for expansion in medium consisting of DMEM/F12 enriched with GlutaMAX and containing glucose, fetal bovine serum, horse serum, penicillin/streptomycin, B27 supplement, epidermal growth factor and basic fibroblast growth factor. After 7-10 days, cultured cells were removed from the flask by trypsinization, seeded onto plastic dishes and incubated at 37°C with 5% CO2. Cells were randomly assigned to experimental groups.

### Method details

#### DNA constructs - Retroviral vector production

##### Retroviral plasmids

To instruct glia-to-neuron reprogramming, we used replication-deficient, self-inactivating retroviral vectors based on the Moloney Murine Leukemia Virus (MoMLV) in order to encode transcription factors (TFs) in the targeted transduced cells as described in our previous studies (Gascón et al., 2016; Heinrich et al., 2010, 2011a, 2014). TFs were expressed under control of an internal compound CAG promoter (that contains the chicken β-actin promoter with the cytomegalovirus early enhancer element and a large synthetic intron optimized for strong and long-term expression (Tashiro et al., 2006; Zhao et al., 2006)) together with DsRed (as fluorescent reporter to visualize transduced cells) located behind an internal ribosomal entry site (IRES) allowing for simultaneous reporter gene expression, as described previously (Heinrich et al., 2010, 2011a). We used the previously described constructs: RV-pCAG-Ascl1-IRES-DsRed (Heinrich et al., 2014) and RV-pCAG-Dlx2-IRES-DsRed (Heinrich et al., 2010, 2011a). We generated a retroviral backbone allowing for polycistronic expression of Ascl1 and Dlx2 (connected via p2A) under control of the CAG promoter together with DsRed: RV-pCAG-Ascl1-p2A-Dlx2-IRES-DsRed. For control experiments, we used the previously described construct encoding DsRed-only driven by the same CAG promoter: RV-pCAG-IRES-DsRed (Heinrich et al., 2010, 2011a).

For RABV-mediated transsynaptic tracing experiments, glial cells were transduced with a retroviral vector (RV-pCAG-DsRedExpress2-2A-G-IRES2-TVA) described previously (Bergami et al., 2015; Deshpande et al., 2013), encoding DsRedExpress2, the RABV glycoprotein (G, from the CVS-11 strain of rabies virus), and the chicken TVA receptor (TVA800: GPI anchored form of TVA). For DREADD-mediated chemogenetic activation of iNs, cortical astroglia were transduced with a retroviral vector (RV-pCAG-EGFP-2A-hM3Dq) described previously (Alvarez et al., 2016) encoding the excitatory Gq-coupled mutated human M3 muscarinic receptor (hM3Dq) and EGFP under the CAG promoter. For optogenetic activation of iNs, glial cells were transduced with a retroviral vector (RV-pUbi-ChR2-EGFP) described previously (Groisman et al., 2020) encoding the Channelrhodopsin2 (ChR2) fused to EGFP under the Ubiquitin promoter.

##### Production of retroviral particles

VSV-G (Vesicular Stomatitis Virus-Glycoprotein)-pseudotyped gamma-retroviral vectors were produced as described in our previous studies (Heinrich et al., 2010, 2011a, 2014) using the MoMLV-based, CAG-driven retroviral expression plasmid and a stable packaging cell line (293GPG) expressing MLV gag-pol and VSV-G under Tet-off control (Ory et al., 1996). Retroviral particles were harvested and concentrated from supernatants of transfected packaging cells by ultracentrifugation following standard protocols, re-suspended in PBS (phosphate-buffered saline), and stored at −80°C until use. Viral titers used for experiments were typically in the range of 10^6^-10^9^ transducing units/mL.

#### Primary cultures of cortical astroglia

For culturing astroglia from the postnatal mouse cerebral cortex we followed the procedures previously described (Heinrich et al., 2010, 2011a). Briefly, C57BL/6J or heterozygous GAD67-GFP mice at the age of postnatal day 5–7 (P5–P7; sex could not be determined at that age) were sacrificed by decapitation and brains were collected in ice-cold HBSS (GIBCO) buffered with 10 mM HEPES (GIBCO). Brains from heterozygous GAD67-GFP mice were identified by GFP expression under a stereomicroscope (Leica M165C). After removal of the meninges, gray matter tissue from the cerebral cortex was dissected and dissociated mechanically. Subsequently, cells were centrifuged for 5 min at 1,000 rpm, re-suspended, and plated in a medium consisting of DMEM/F12 enriched with GlutaMAX (GIBCO), 3.5 mM glucose (Sigma), 10% fetal bovine serum (GIBCO), 5% horse serum (GIBCO), penicillin/streptomycin (GIBCO) and supplemented with B27 (GIBCO), 10 ng/mL human epidermal growth factor (EGF, GIBCO) and 10 ng/mL human basic fibroblast growth factor (bFGF, GIBCO). Cells were harvested after 7-10 days using trypsin/EDTA (GIBCO) and plated onto plastic dishes (diameter: 3.5 cm) in the same medium. The vast majority of the cells (> 90%) in these cultures were positive for glial fibrillary acidic protein (GFAP) as previously described (Berninger et al., 2007; Gascón et al., 2016; Heinrich et al., 2010, 2011a). Retroviral transduction of astroglia was performed using VSV-G-pseudotyped retroviral vectors 3-4h after plating. Twenty-four hours after transduction, the transduction medium was completely removed and the cells washed several times with PBS before being grafted into the hippocampus of MTLE-HS mice and allowed for neuronal reprogramming *in vivo* (see below). In all experiments, cells were randomly assigned to experimental groups (i.e., transduction with the different retroviruses).

#### Induction of MTLE-HS in adult mice

##### Selection of the model of MTLE-HS

We used a well-established mouse model of chronic MTLE-HS that has been extensively described (e.g., Arabadzisz et al., 2005; Duveau et al., 2016; Heck et al., 2004; Heinrich et al., 2006, 2011b; Janz et al., 2017; Maroso et al., 2010; Müller et al., 2009; Nitta et al., 2008; Pernot et al., 2011; Riban et al., 2002; Stamboulian-Platel et al., 2016; for review, see Löscher et al., 2020). We selected this model as one used by the NIH/NINDS Epilepsy Therapy Screening Program to test efficacy of new antiepileptic treatments (Löscher et al., 2020) given the poor responsiveness of seizures to various antiepileptic drugs (Duveau et al., 2016; Löscher et al., 2020; Riban et al., 2002), thus mimicking drug-resistant seizures as described in MTLE-HS patients (Cendes et al., 2014; Engel, 2001). Importantly, previous continuous EEG-monitoring revealed high-frequency of non-convulsive EEG seizures comparable between MTLE-HS mice, as well as stable and reproducible seizure baseline over time in each MTLE-HS mouse, which render this model suitable for reliable assessment of antiepileptic strategies (Duveau et al., 2016; Löscher et al., 2020; Maroso et al., 2010).

##### Intrahippocampal injection of kainate (KA)

Adult C57BL/6J male mice (Charles River, France; 2-3 months of age; 25-30 g) or heterozygous GAD67-GFP male mice (Tamamaki et al., 2003) (2-3 months of age; 25-30 g) were injected with KA unilaterally in the dorsal hippocampus as previously described (Heinrich et al., 2006, 2011b; Riban et al., 2002). Briefly, mice first received a premedication with Xylazine (Rompun®, 5-10 mg/kg i.p., Bayer) inducing sedation, muscle relaxation and analgesia, before being placed in an isoflurane induction chamber (5% isoflurane (Vetflurane, Virbac), 95% air) until the animals were fully anaesthetized. While being maintained under isofluorane-induced anesthesia (2% isoflurane), mice were positioned in a stereotaxic apparatus in flat skull position. A small unilateral craniotomy (diameter: 0.5 mm) was drilled at the following coordinates: anteroposterior (AP) = −2 mm, mediolateral (ML) = −1.5 mm with bregma as reference. A stainless steel cannula (outer diameter: 0.28 mm) connected to a 0.5 μL micro-syringe (Hamilton, Bonaduz, Switzerland) via PE20 tubing containing distilled water, was filled with a 20 mM solution of KA (Sigma-Aldrich, France) in 0.9% sterile NaCl, and inserted in the right dorsal hippocampus at the following coordinates: AP = −2, ML = −1.5, dorsoventral (DV) = −2 mm with bregma as reference. Mice were injected with 50 nL (i.e., 1 nmol) of the KA solution during 1 min using a micro-pump (CMA/100, Carnegie Medicin, Sweden) operating the micro-syringe as previously described (Heinrich et al., 2006, 2011b). After injection, the cannula was left in the hippocampus for additional 2 min to avoid reflux along the cannula track. Control mice were injected with 50 nL of 0.9% sterile NaCl under the same conditions. Mice were sutured and maintained on a warming pad until complete recovery from anesthesia. In all experiments, mice were randomly assigned to experimental groups (i.e., injection of saline or KA).

##### Validation of KA-induced status epilepticus

After recovery from anesthesia, animals were visually inspected for up to 10h to determine their behavior during the initial KA-induced status epilepticus. It has been well characterized that intrahippocampal KA injection initially induces in all injected mice a limbic and non-convulsive status epilepticus associated with stereotyped behaviors of the animals concomitant with EEG alterations (Löscher et al., 2020; Maroso et al., 2010; Riban et al., 2002). All KA-injected mice included in this study displayed a comparable and typical behavior of status epilepticus as previously described, including mild asymmetric clonic movements of the forelimbs, clonic deviations of the head, rotations, prolonged periods of immobilization/prostration and occasional bilateral clonic seizures, in agreement with previous reports (Heinrich et al., 2006, 2011b; Riban et al., 2002). Importantly, previous studies demonstrated with chronic EEG monitoring that all KA-treated mice exhibiting this typical behavioral pattern of status epilepticus: i) show a comparable epileptogenesis with a similar time-course of ∼2 weeks after KA injection, and develop spontaneous recurrent seizures in the hippocampus during the chronic phase of the disease, and ii) display a typical pattern of hippocampal sclerosis (Heinrich et al., 2011b; Riban et al., 2002).

#### Grafting of transduced-astroglia in MTLE-HS mice

Twenty-four hours after retroviral transduction, transduced-astroglia were extensively washed several times with sterile PBS to completely remove potentially residual viral particles, before being harvested using trypsin/EDTA and centrifuged for 5 min at 1,200 rpm at 4°C. Supernatant was carefully discarded and the cell pellet was washed with fresh 0.9% sterile NaCl before additional centrifugation (5 min; 1,200 rpm; 4°C). The cell pellet was gently re-suspended in the residual volume following supernatant removal and kept on ice for 5-10 min until intrahippocampal transplantation. Five days after KA injection, MTLE-HS mice (C57BL/6J wild-type mice) were anaesthetized and ∼10,000 cells (0.5-1 μL) were stereotaxically transplanted during 15 min in the dentate gyrus (using a cannula and a micro-syringe as described above) at the following coordinates: AP = −2, ML = −1.5, DV = −2 mm with bregma as reference. Of note, we used a comparable time-window post KA-induced lesion for grafting, as previously reported in other experimental settings for grafting in the lesioned adult mouse visual or motor cortex (Espuny-Camacho et al., 2018; Michelsen et al., 2015). Mice were sutured and maintained on a warming pad until complete recovery from anesthesia. In all experiments, mice were randomly assigned to experimental groups (i.e., grafting of astroglia transduced with the different retroviruses).

#### Retroviral injection in MTLE-HS mice

To instruct neuronal reprogramming of endogenous hippocampal reactive glial cells proliferating in the sclerotic hippocampus, a retrovirus encoding Ascl1 and Dlx2 together with DsRed, or DsRed-only for control, was injected in the hippocampus of MTLE-HS mice (C57BL/6J or heterozygous GAD67-GFP) at 5 dpKA following similar procedures as for KA injection. For optogenetic activation of iNs, mice received a retrovirus encoding Ascl1/Dlx2 (DsRed) and a retrovirus encoding ChR2 (GFP). Briefly, a cannula connected to a 2 μL micro-syringe via PE20 tubing (containing distilled water) was filled with the retroviral suspension and inserted in the dentate gyrus at the following coordinates: AP = −2, ML = −1.5, DV = −2 mm with bregma as reference. The viral suspension (0.5-1 μL) was slowly injected during 30-40 min to allow for an optimal diffusion of viral particles inside the dentate gyrus. After injection, the cannula was left in the hippocampus for additional 5 min to avoid reflux along the cannula track and was then slowly removed. Mice were sutured and maintained on a warming pad until complete recovery from anesthesia. In all experiments, mice were randomly assigned to experimental groups (i.e., injection of the different retroviruses).

#### Bromodeoxyuridine (BrdU) treatment

To examine germinal activity in the dentate subgranular zone after KA or saline injection, mice were treated with the thymidine analog Bromodeoxyuridine (BrdU, Sigma Aldrich) in drinking water (1 mg/mL BrdU supplemented with 1% sucrose) for 3, 5, or 7 consecutive days starting immediately after intrahippocampal KA or saline injection until sacrifice.

To determine which cell types proliferate in the KA-injected hippocampus at the time of retrovirus injection, mice received a short pulse of BrdU (i.p.; 100 mg/kg in saline) at 5 dpKA (BrdU pulse protocol). Alternatively, KA-injected mice received BrdU in drinking water during a 2-day time-window (between 3 and 5 dpKA; 1 mg/mL BrdU supplemented with 1% sucrose) prior to retrovirus injection (BrdU drinking water protocol). In addition, these two BrdU labeling protocols were also employed to initially label dividing hippocampal reactive glia that were subsequently reprogrammed into iNs.

#### Rabies virus-mediated monosynaptic tracing

To identify the presynaptic partners projecting onto iNs derived from endogenous hippocampal glia or from grafted astroglia, we used a G-deleted, GFP-expressing, EnvA-pseudotyped rabies virus (ΔG RABV) as previously described (Bergami et al., 2015; Deshpande et al., 2013; Wickersham et al., 2007). Construction of the G-deleted, GFP-expressing RABV (SADΔG-GFP) as well as pseudotyping of SADΔG-GFP with the envelope protein EnvA have been described before (Wickersham et al., 2007). To identify the presynaptic partners projecting onto iNs, cultured astroglia or resident hippocampal glia were co-transduced with a retrovirus encoding G and TVA (i.e., the EnvA receptor) as well as a retrovirus encoding the reprogramming TFs. 6-8 weeks after retroviral delivery, mice were injected with the GFP-encoding, EnvA-pseudotyped ΔG RABV (0.5 μL) and sacrificed 1 week later.

#### Electrophysiology

##### Slice preparation

MTLE-HS mice, in which reprogramming of glia had been induced *in situ* or following grafting, were used for electrophysiological recordings at 2-3 mpi. Mice were deeply anesthetized with isoflurane (Forane, Abbvie), decapitated and the brains were quickly collected into a chilled artificial cerebrospinal fluid (ACSF; Composition in mM: NaCl, 85; Sucrose, 73; KCl, 2.5; NaHCO_3_, 25; CaCl_2_, 0.5; MgCl_2_, 7; NaH_2_PO_4_, 1.25 and glucose, 10) saturated with 95% O_2_ and 5% CO_2_ (pH 7.4). Coronal brain slices (250 μm thick) containing the dorsal hippocampus were prepared using a vibratome (VT1200 S, Leica) and transferred to standard ACSF (Composition in mM: NaCl, 125; KCl, 2.5; NaHCO_3_, 25; CaCl_2_, 2; MgCl_2_, 1; NaH_2_PO_4_, 1.25 and glucose, 12; pH 7.4). Slices were incubated in standard ACSF at 34°C for 1h followed by one additional hour at room temperature (21°C ± 2°C). For recordings, individual slices were transferred into a recording chamber mounted on the stage of an upright microscope (Slice Scope Pro 6000 System, Scientifica, UK). Slices were constantly perfused at the rate of 1-2 mL/min with standard ACSF maintained at 30°C and saturated with 95% O_2_ and 5% CO_2_. Cells were visualized using a 40X (0.8 numerical aperture) water immersion objective with infrared DIC videomicroscopy.

##### Electrophysiological recordings

Whole-cell patch-clamp recordings were performed at 30°C ± 2°C (in-line Peltier heater, Scientifica, UK) using recording pipettes (5-10 MΩ) prepared from borosilicate glass capillaries (1B150F-4, World Precision Instruments) and filled with a solution having the following composition (in mM): K-gluconate, 125; NaCl, 5; Na_2_-ATP, 2; MgCl_2_, 2; EGTA, 1; HEPES, 10, and biocytin (10 mM) to allow for subsequent morphological analysis (pH 7.4; osmolarity, 280 mOsm). In some cases, Alexa 647 hydrazide (100 μM, Invitrogen) was also added to the pipette solution for direct visualization of the recorded cells. Current- and voltage-clamp recordings were performed using a MultiClamp 700B amplifier (Molecular Devices), digitized (Digidata 1550B, Molecular Devices), and acquired at 20 kHz onto a computer using pClamp 10.7 software (Molecular Devices) which was also used for offline data analysis. Criteria to include cells in the analysis were the following: i) seal resistances between 4 and 18 GΩ, ii) initial series resistance was less than 40 MΩ and did not change by more than 20% throughout the recording period. The numbers of cells recorded for each condition are indicated in the Figure legends.

iNs were visually identified on the basis of their fluorescence emission: DSRED+ (Ascl1/Dlx2) and GFP+ (ChR2, see below) using appropriate LED excitation (CoolLED pE-100) and emission filters, and were selected according to their neuronal morphology. Digital pictures of the recorded cells were acquired using a digital camera. In current-clamp recordings, the membrane potential of iNs was kept at −60 mV by passing a holding current. Passive and active membrane properties were recorded by applying a series of hyperpolarizing and depolarizing current steps (10 pA steps, 500 ms). To examine the occurrence of spontaneous excitatory synaptic events, cells were kept in voltage-clamp at a holding potential of −70 mV.

##### Optogenetic activation of iNs and recording of responses in GCs

To assess whether iNs establish GABAergic synapses onto GCs, we followed an optogenetic strategy based on selective expression of channelrhodopsin2 (ChR2) in iNs, thereby allowing their blue light-mediated activation while recording responses in GCs. We simultaneously targeted expression of the reprogramming genes Ascl1/Dlx2 (DsRed) and ChR2 (GFP) to hippocampal reactive glia through direct retrovirus injection *in situ* at 5 dpKA. Alternatively, astroglia were co-transduced *in vitro* with retroviruses encoding respectively Ascl1/Dlx2 (DsRed) and ChR2 (GFP), shortly before (24h) being grafted in the hippocampus of MTLE-HS mice at 5 dpKA.

To assess synaptic input in GCs, experiments were performed in current-clamp mode. Photostimulation of ChR2+ iNs was achieved with blue light pulses (laser 473 nm; duration, 100 ms; laser power, 4 mW) using a Laser Applied Stimulation and Uncaging (LASU) system (Scientifica). The laser output was driven by a transistor-transistor logic (TTL) output from the Clampex software of the pClamp 10.7 program suite (Molecular Devices). For light-evoked depolarization of iNs, a laser spot was directed to the cell soma. GCs were selected for recording according to their morphology and position within the GC layer, as well as their vicinity to GFP+ (i.e., ChR2+) iN processes. For GC recordings, a raster of 473 nm blue light was applied on top of the cell to cover the adjacent GFP+ processes. For every GC, we recorded 10-15 consecutive trials of photostimulation delivered every 10 s. To assess the reversal potential of the response, the membrane potential of GCs was kept at different values by passing a holding current. The reversal potential for Cl^-^ ions was calculated using the Nernst equation. In our experimental conditions, E_Cl_^−^ was −69.8 mV. To determine the nature of the synaptic responses, iNs were activated by blue light as described above, and we assessed whether responses could be abolished in the presence of gabazine (SR 95531 hydrobromide, 10 μM, Tocris), a selective antagonist of GABA_A_ receptors located at the synaptic cleft (Wlodarczyk et al., 2013).

#### Electroencephalographic (EEG) recordings

##### Electrode implantation

MTLE-HS mice were anaesthetised and implanted with electrodes as described previously (Heinrich et al., 2011b; Riban et al., 2002). Briefly, mice were implanted in the injected hippocampus (at the same coordinates as for injection) with a bipolar electrode made of two twisted enamel insulated stainless steel wires (diameter = 170 μm, distance between the tips = 0.4 mm), each of them being soldered to a male connector (Farnell, France). One additional small hole was drilled above the cerebellum, in which a reference monopolar electrode made of a tungsten wire (diameter = 250 μm) soldered to a male connector, was inserted so that only the tip (0.2 mm) protruded onto the cerebellar tissue. Connectors were fixed on the skull with dental acrylic cement and mice were maintained on a warming pad until complete recovery from anesthesia.

##### Intrahippocampal EEG recordings

EEG activity (i.e., local field potentials) was recorded using a digital acquisition computer-based system (Coherence, Natus Deltamed, France; sampling rate 1024 Hz) in freely moving mice placed in Plexiglas test chambers within a Faraday cage, as described previously (Heinrich et al., 2011b; Riban et al., 2002). All EEG monitoring sessions were performed between 1:00 and 5:00 p.m. as follows. Mice were first connected to the recording system by plugging male connectors into female connectors (Farnell, France) and were habituated to their recording chamber for at least 1h before hippocampal activities were recorded for 2-3h as previously described (Heinrich et al., 2011b; Riban et al., 2002; Stamboulian-Platel et al., 2016). For each experiment, mice with different treatments (i.e., controls, mice with *in situ* iNs, or graft-derived iNs) were randomly assigned to EEG monitoring sessions performed several times over a week. Importantly, previous studies with continuous EEG monitoring in this model revealed high-frequency of non-convulsive electrographic seizures similar between MTLE-HS mice, as well as a stable and reproducible number of spontaneous recurrent seizures (per hour) during day time in each MTLE-HS mouse (Duveau et al., 2016; Löscher et al., 2020; Maroso et al., 2010). Consistent with these data, periods of 2-3h of EEG recordings were previously shown to allow for reliable quantification of the number and duration of hippocampal seizures in MTLE-HS mice, and periods of 2-3h were classically used in previous studies to evaluate efficacy of potential antiepileptic treatments (Maroso et al., 2010; Riban et al., 2002; Stamboulian-Platel et al., 2016). A referential setup was used in which hippocampal electrodes were referenced with the electrode placed over the cerebellum. At the end of the experiments, we confirmed in all mice the correct position of the electrodes in the hippocampus using hippocampal sections.

#### Selective chemogenetic activation of iNs

To assess the impact of selective activation of GABAergic iNs on hippocampal seizures, astroglia were simultaneously co-transduced *in vitro* with a retrovirus encoding the excitatory Gq-coupled mutated human M3 muscarinic receptor (GFP) together with a retrovirus encoding Ascl1 and Dlx2 (DsRed), shortly before (24h) being grafted in the hippocampus of MTLE-HS mice at 5 dpKA. To study the effects of the activation of hM3Dq+ GABAergic iNs on seizures, grafted MTLE-HS mice were EEG monitored at 3–4 mpi for 2h of reference recording before being injected intraperitoneally (i.p.) with CNO (clozapine-N-oxide, 20 mg/kg, diluted in 2% DMSO/saline). After recovery from handling and i.p. injection (∼15 min), mice were EEG monitored again for 2h. Dosing and timing of CNO were defined based on previous reports assessing seizure activity in epileptic animals (Kätzel et al., 2014; Smith et al., 2016). For additional control, EEGs were acquired in non-grafted MTLE-HS mice before and after injection of the same dose of CNO to assess potential effects of CNO on its own on seizure activity. Importantly, similar doses of CNO were previously used successfully without any non-specific effects on behavior and neuronal activity (Mahler et al., 2014; Smith et al., 2016). It was previously shown that CNO on its own and its potential off-targets do not interfere with focal seizure activity compared to control vehicle treatment in chemoconvulsant-induced epilepsy models (Kätzel et al., 2014). Of note, seizure activity was previously reported to be significantly reduced in focal seizure models already within 10 min after i.p. injection of CNO targeting inhibitory DREADDs expressed in excitatory neurons (Kätzel et al., 2014; Smith et al., 2016).

#### Immunohistochemistry

Immunostainings were performed as described previously (Heinrich et al., 2010, 2011a, 2014). Briefly, mice were anesthetized and transcardially perfused with 4% PFA in PBS for 30 min. The brains were removed from the skull and post-fixed in 4% PFA for 4h at 4°C and subjected to cryoprotection in 20% sucrose (Sigma) in PBS overnight at 4°C. The brains were cut in coronal sections of 40-50 μm on a cryostat and collected in PBS. Sections were used for immunohistochemistry using a free-floating procedure. Sections were washed three times in PBS (15 min each; pH 7.4) and were first pre-treated in 0.25% Triton X-100 in PBS for 30 min followed by incubation in 2% bovine serum albumin (BSA) in PBS for 30 min. Primary antibodies were incubated on specimens for 90 min at room temperature followed by overnight incubation at 4°C in 2% BSA, 0.1% Triton X-100 in PBS. After extensive washing in PBS, sections were incubated with appropriate species-specific secondary antibodies conjugated to fluorochromes or biotin for 2h in the dark at room temperature followed by extensive washing in PBS. Following treatment with secondary antibodies conjugated to biotin, sections were incubated for 2h at room temperature with Alexa Fluor 405-streptavidin or Alexa Fluor 647-streptavidin. Finally, tissue sections were mounted onto glass slides, air-dried, and coverslipped with an anti-fading mounting medium (Aqua Poly/Mount; Polysciences, Warrington, PA). A list of the primary antibodies is included in the Key resources table.

For BrdU immunohistochemistry, staining of other antigens was performed first and sections were subsequently fixed with 4% PFA (30 min). Sections were then pre-treated with 2N HCl for 30 min and subsequently neutralized with sodium-tetraborate (Na_2_B_4_O_7_, 0.1M, pH 8.5) during 2 X 15 min before being stained for BrdU.

For biocytin staining, slices were fixed after patch-clamp recording by immersion in 4% PFA in PBS for 24h. After washing in PBS, slices were blocked with 0.5% BSA in PBS for 1h and incubated in a solution containing 0.3% Triton X-100 with Alexa Fluor 647-streptavidin for 2h.

#### Confocal microscopy

Immunostainings were analyzed with laser-scanning confocal microscopes (LSM 710 and LSM 880, Carl Zeiss, Germany). Super-resolution images of axons and synaptic boutons from graft-derived iNs were acquired using the Airyscan technology (Zeiss). Z stacks of digital images were captured using the ZEN 2.3 software (Zeiss). For the figures, the Z stacks were collapsed in one resulting picture using the maximum intensity projection function provided by the software. Alternatively, when appropriate, single plane confocal images were extracted from the Z stacks. In order to acquire dentate gyrus overviews with high resolution, the Tile Scan module (ZEN 2.3 software, Zeiss) was used to scan areas that exceeded the size of an individual image field. Multiple individual images overlapping each other were acquired and automatically stitched together into a single composite image (15% overlap for optimal stitching). These composite images are indicated in the Figure Legends.

### Quantification and statistical analysis

Following confocal microscopy, Z stacks of digital images were analyzed for cell quantifications using ImageJ 1.51v software (National Institute of Health, USA). Cell counts were done by moving through the Z stacks of confocal images, which allowed for accurate visualization of the cells of interest. Hippocampal EEG seizures were quantified using the Coherence software (Natus Deltamed, France). Electrophysiological recordings were analyzed with pClamp 10.7 software (Molecular Devices). Throughout the manuscript, graphs represent mean ± SEM. The *n* values represent the number of mice. Animals were randomly assigned to groups. Statistical analysis was performed with GraphPad Prism v.8 software. The sample sizes in the present study are comparable to those reported in previous studies in the field of *in vivo* reprogramming (e.g., Gascón et al., 2016; Grande et al., 2013; Guo et al., 2014; Heinrich et al., 2014; Matsuda et al., 2019; Mattugini et al., 2019; Niu et al., 2013; Torper et al., 2015) and in previous reports to assess efficacy of antiepileptic strategies (e.g., Baraban et al., 2009; Henderson et al., 2014; Kätzel et al., 2014; Maroso et al., 2010; Upadhya et al., 2019). Quantification procedures and statistical tests used for every analysis are indicated in detail below. For statistical comparisons, differences were considered statistically significant when the p value was < 0.05. Statistical significance was defined at ^∗^p < 0.05, ^∗∗^ p < 0.01, ^∗∗∗^ p < 0.001 and ^∗∗∗∗^ p < 0.0001. For every experiment, statistical tests and significance as well as *n* values are indicated in detail below and in the Figure Legends.

#### Thickness of the granule cell layer

For each mouse, the thickness (μm) of the suprapyramidal granule cell layer was measured in its largest portion on at least three sections both in the contralateral and KA-injected hippocampus at the level of the injection site, where granule cell dispersion was reported to be the most pronounced (Heinrich et al., 2011b). Five mice were quantified and data are expressed as mean thickness (μm) ± SEM. Statistical analysis was performed using a two-tailed Mann-Whitney test (Figure S1D).

#### Loss of endogenous GABAergic interneurons

To determine the extent of GABAergic neuron loss in MTLE-HS mice, we used GAD67-GFP mice and quantified the number of GFP+ GABAergic interneurons in the contralateral and KA-injected hippocampus. For each mouse, the surface of the contralateral and KA-injected hippocampus was delineated using ImageJ on three Z stacks imaged at the level of the injection site. The number of GFP+ interneurons was counted within the respective hippocampal area moving through the Z stacks and expressed as GFP+ neuron numbers per mm^3^. Four mice were quantified at 5 dpKA and values are given as mean numbers ± SEM. Statistical analysis was performed using a two-tailed Mann-Whitney test (Figure S1F).

#### BrdU+ / DCX+ cells in the subgranular zone

Using ImageJ, the subgranular zone area was carefully delineated on digital images and its surface was calculated. For each saline- or KA-injected mouse, we counted the number of BrdU/DCX-double positive cells in the subgranular zone area at 3d (saline, n = 4; KA, n = 3), 5d (saline, n = 4; KA, n = 4) and 7d (saline, n = 4; KA, n = 3) post injection. For comparison between mice, the number of BrdU/DCX+ cells was expressed per mm^2^ for each mouse. For each group of mice, data are expressed as mean numbers ± SEM. Statistical analysis was performed based on two-way ANOVA (“Treatment”: saline or KA; “Time post injection”: 3, 5 or 7 days post injection) followed by Sidak’s multiple comparison *post hoc* tests for defining statistical differences between groups (Figure S3C).

#### BrdU+ glial cells in the dentate gyrus

To quantify which cell types proliferate in the KA-injected dentate gyrus at the time of retrovirus injection, mice received a short pulse of BrdU at 5 dpKA. We counted the number of BrdU+ cells and the number of BrdU/GFAP+, BrdU/IBA1+, BrdU/OLIG2+ and BrdU/DCX+ cells. For each mouse, the number of BrdU+ cells expressing each marker was expressed as percentage of the number of BrdU+ cells. Four mice were analyzed and values are given as mean percentages ± SEM (Figure S3F).

To determine which proliferating cells were both labeled by BrdU and transduced by a control retrovirus, mice were treated with BrdU following two different BrdU protocols (BrdU pulse or BrdU drinking water, see above) and were injected with the control retrovirus (DsRed) at 5 dpKA. First, we counted the number of DSRED/BrdU-double labeled cells following each BrdU protocol. For each mouse, numbers were expressed as percentage of the total number of DSRED+ transduced cells. For each group of mice (BrdU pulse, n = 4; BrdU drinking water, n = 4), data are expressed as mean percentages ± SEM (Figure S3L). Second, to characterize the identity of the DSRED/BrdU+ cells, the number of cells expressing DCX, NEUN, OLIG2, IBA1 or GFAP were quantified in each mouse and expressed as percentage of DSRED/BrdU+ cells. For each group of mice (BrdU pulse, n = 4; BrdU drinking water, n = 3), values are given as mean percentages ± SEM (Figure S3N).

#### Proliferating cells transduced by a retrovirus

To determine which dividing hippocampal cells are transduced by a retrovirus, mice received the control retrovirus encoding DsRed-only at 5 dpKA. The number of DSRED+ transduced cells expressing GFAP, OLIG2, IBA1, DCX or NEUN were quantified in each mouse and expressed as percentage of the number of DSRED+ cells. For each group of mice, values are given as mean percentages ± SEM; Figure 2C: glial markers (n = 3), neuronal markers (n = 5); Figure S3I: 4 dpi (n = 7), 6 wpi (n = 3). Statistical analysis was performed using a two-tailed Mann-Whitney test (Figure 2C).

#### Glia-to-neuron reprogramming efficiency

To determine the number of iNs, we counted for each mouse the number of DSRED+ transduced cells and the number of DSRED+ cells expressing neuronal markers (DCX, MAP2, NEUN). The number of DSRED+ iNs was expressed as percentage of the total number of DSRED+ cells (i.e., reprogramming efficiency). For each condition (Control, Ascl1, Ascl1/Dlx2), several mice were quantified and values are given as mean percentages ± SEM (Figure 1F: control (n = 4), Ascl1 (n = 4), Ascl1/Dlx2 (n = 4); Figure 2H: control (n = 5), Ascl1/Dlx2 (n = 6); Figure S2F: control (n = 4), Ascl1/Dlx2 (n = 4); Figure 2E, DCX: control (n = 3), Ascl1/Dlx2 (n = 5) and NEUN: control (n = 3), Ascl1/Dlx2 (n = 6)). Statistical analysis was performed using a two-tailed Mann-Whitney test (Figures 1F, 2E, 2H, and S2F).

In control experiments, the number of DSRED+ cells transduced with the control retrovirus (DsRed-only) and expressing glial markers (GFAP, OLIG2 or IBA1) or neuronal markers (DCX, MAP2 or NEUN) was quantified in each mouse and expressed as percentage of the number of DSRED+ cells. For each group of mice, values are given as mean percentages ± SEM (Figure 1D: astrocyte marker (n = 4), neuronal markers (n = 6); Figure 2C: glial markers (n = 3), neuronal markers (n = 5)). Statistical analysis was performed using a two-tailed Mann-Whitney test (Figures 1D and 2C).

#### BrdU labeling of glia-derived iNs

To demonstrate that hippocampal reactive glia-derived iNs were indeed generated *de novo*, we initially labeled dividing glial cells with BrdU prior to, or at the time of Ascl1/Dlx2-retrovirus injection using two different BrdU protocols (BrdU pulse or BrdU drinking water, see above). For each mouse, the number of DSRED/NEUN+ iNs immunoreactive for BrdU was quantified and expressed as percentage of the number of DSRED/NEUN+ iNs. For each group of mice (BrdU pulse, n = 4; BrdU drinking water, n = 4), values are given as mean percentages ± SEM (Figure 2K).

#### Phenotype of glia-derived iNs

To determine the phenotype of glia-derived iNs, we used GAD67-GFP mice from which glia-derived GABAergic iNs will turn on GFP under control of the GAD67 promoter. For each mouse, the number of DSRED/NEUN+ iNs immunoreactive for GFP was counted and expressed as percentage of the total number of DSRED/NEUN+ iNs. For each condition (Ascl1 or Ascl1/Dlx2), values are given as mean percentages ± SEM; Figure 1I: Ascl1 (n = 4), Ascl1/Dlx2 (n = 4); Figure 2N: Ascl1/Dlx2 (n = 5). Statistical analysis was performed using a two-tailed Mann-Whitney test (Figure 1I).

To determine whether iNs expressed interneuron subtype markers, sections were immunostained for DSRED and NEUN (to identify glia-derived iNs) and for one subtype marker at a time. For each mouse, the number of DSRED/NEUN+ iNs expressing either Vasoactive Intestinal Peptide (VIP), somatostatin (SST), neuropeptide Y (NPY), parvalbumin (PV) or calretinin (CALB2) was quantified, and expressed as percentage of the number of DSRED/NEUN+ iNs counted in each respective staining condition. For each group of mice (graft-derived iNs, Figure 1L: VIP, SST, CALB2 (n = 4 each); *in situ* iNs, Figure 2Q: VIP (n = 5), NPY (n = 5), SST (n = 7)), values are given as mean percentages ± SEM. Of note, we did not find graft-derived iNs reliably expressing NPY or PV. *In situ* iNs were not find to express PV or CALB2.

To determine whether iNs derived from hippocampal glia expressed PROX1, the number of DSRED/NEUN+ iNs expressing PROX1 was counted for each mouse and expressed as percentage of the number of iNs. Data are expressed as mean percentage ± SEM (n = 4).

#### C-FOS immunoreactivity: marker of iN activity

To evaluate whether iNs were physiologically active, we counted for each mouse the number of DSRED/NEUN+ iNs exhibiting C-FOS+ nuclei that was expressed as percentage of the total number of DSRED/NEUN+ iNs. For each group of mice (*in situ* iNs, n = 3, Figure 5G; graft-derived iNs, n = 3 without CNO, n = 4 after CNO treatment, Figure 6C), values are given as mean percentages ± SEM.

#### RABV-mediated synaptic tracing of iN connectivity

To quantify the number of presynaptic partners sending projections onto iNs, we counted for each mouse the number of GFP+ presynaptic neurons (i.e., RABV-only transduced neurons) and double-transduced iNs (i.e., GFP+/DSRED+ corresponding to starter iNs). For comparison between mice, the number of GFP+ presynaptic cells was normalized on number of starter neurons (i.e., connectivity ratio) for each mouse to take into account variations in the number of starter neurons. For each mouse, the numbers of GFP+ presynaptic partners encountered in distinct brain areas were expressed as connectivity ratios. For each group of mice (*in situ* iNs (n = 3); graft-derived iNs (n = 3)), values are given as mean connectivity ratios ± SEM (Figures 3E and S4E).

#### Tracing of territories innervated by iNs

To assess the spatial extent of territories innervated by iNs, Z stacks of representative examples were used for tracing with Neurolucida 360® DSRED+ neuronal processes originating from iNs and extending along the rostro-caudal axis of the hippocampus using consecutive adjacent sections. Subsequently, the Neurolucida fiber traces were superimposed onto drawings of the respective hippocampal sections (Figures 3F and S4F).

#### Electrophysiology

Input resistance (Rin) was calculated from the peak of the voltage response to a −20 pA, 500 ms current step according to Ohm’s law. The resting membrane potential (mV) was assessed immediately after break-in by reading the voltage value in the absence of current injection (I = 0 configuration). The membrane capacitance (Cm, in pF) was obtained with the following equation: membrane time constant (τm) = Rin X Cm, with τm being derived from single exponential fitted to voltage response to a −20 pA, 500 ms current step. Action potential amplitude (mV) was measured on the first spike observed at rheobase, from threshold to positive peak. Firing frequency (Hz) was calculated dividing the number of spikes by the step duration. Four iNs derived from hippocampal glia (n = 3 mice) and four iNs derived from grafted glia (n = 4 mice) were analyzed (Figures S5A–S5E).

#### Spontaneous recurrent hippocampal seizures

Digital EEG recordings were analyzed with the same software as for acquisition (Coherence, Natus Deltamed, France) by an investigator blind to the treatment of the mice and by using the bipolar hippocampal derivation as described previously (Arabadzisz et al., 2005; Heinrich et al., 2011b; Riban et al., 2002). To assess the impact of iNs on seizure activity during the chronic phase, we quantified the number and duration of non-convulsive EEG seizures occurring in the KA-injected hippocampus on each EEG recording. Previous work in this model defined spontaneous recurrent seizures as “hippocampal paroxysmal discharges” mostly consisting of slow rhythmic high-voltage sharp waves (3-5 Hz) followed by higher-frequency and lower-amplitude spikes (10-14 Hz) (Arabadzisz et al., 2005; Heinrich et al., 2011b; Maroso et al., 2010; Riban et al., 2002). In agreement with these previous reports, a hippocampal seizure was defined as a paroxysmal discharge lasting ≥18 s. Sharp waves and spikes were defined with amplitude at least two-fold above the EEG background activity. Two sharp waves/spikes belonged to the same seizure if the time interval between these two sharp waves/spikes was < 1 s.

##### Impact of hippocampal glia-derived iNs on seizures

To assess whether GABAergic iNs alter seizure activity during the chronic phase, we quantified for each control MTLE-HS mouse (i.e., injected with the control retrovirus; n = 6) and each MTLE-HS mouse with iNs (i.e., injected with the Ascl1/Dlx2-encoding retrovirus; n = 6) the number and duration (s) of non-convulsive EEG hippocampal seizures and calculated their cumulative duration (i.e., time spent in seizures in min). For each group of mice, data are expressed as mean values per hour ± SEM. Statistical analysis was performed using a two-tailed Mann-Whitney test (Figures 5D and 5E).

##### Impact of grafted glia-derived iNs on seizures

To assess the impact of graft-derived GABAergic iNs on chronic seizure activity, EEG recordings from grafted MTLE-HS mice were analyzed before and after CNO-mediated activation of iNs, and compared to control non-grafted MTLE-HS mice before and after CNO treatment. We quantified for each control MTLE-HS mouse (n = 4) and for each MTLE-HS mouse with iNs (i.e., derived from grafted Ascl1/Dlx2-transduced astroglia; n = 5) the number of non-convulsive EEG hippocampal seizures before and after CNO treatment. For each group of mice, values are expressed as average numbers of seizures per hour ± SEM. Statistical analysis was performed using repeated-measures two-way ANOVA using two factors: 1) Presence of iNs: “no iNs in control MTLE-HS mice” or “MTLE-HS mice with iNs”, F_1,7_ = 16.38, p = 0.0049; 2) CNO treatment: “before CNO” or “post CNO”, F_1,7_ = 11.57, p = 0.0114. Differences between individual groups were then assessed by Sidak’s multiple comparison *post hoc* analyses (Figure 6G).

To assess the impact of CNO-induced activation of iNs on remaining EEG seizure activity (i.e., that had not been eliminated by iNs in absence of CNO), we performed pairwise comparisons of ‘seizure numbers’ and ‘time in seizures’ before and after CNO in the same MTLE-HS mice with hM3Dq+ GABAergic iNs (n = 5; same mice as in Figure 6G, red bars). For each mouse, the number of EEG seizures and their cumulative duration (i.e., time spent in seizures in min) were calculated per hour before and after CNO, and were expressed as percentage of the values before CNO (set at 100%). Values are given as mean percentages ± SEM. Pairwise comparisons of ‘seizure numbers’ and ‘time in seizures’ were also performed before and after CNO treatment in control non-grafted MTLE-HS mice (n = 4; same mice as in Figure 6G, white bars). Statistical analysis was performed using a Wilcoxon matched-pairs test (Figures 6H, 6I, S6F, and S6G).

#### Extent of neuronal loss in CA1

At the end of the experiments, we assessed the extent of KA-induced neuronal loss in the CA1 hippocampal subfield in the EEG-monitored MTLE-HS mice injected with the control (n = 6) or the Ascl1/Dlx2-encoding retrovirus (n = 6). For each mouse, we carefully delineated using ImageJ the CA1 region in both the contralateral (non-injected) and KA-injected hippocampus on NEUN-stained sections. Digital images were acquired by using identical acquisition settings at the confocal microscope to allow comparison between samples. Using ImageJ, we calculated the mean fluorescence intensity of NEUN staining (or mean gray value, i.e., the sum of the gray values of all pixels within the selected area divided by the number of pixels), which reflects the density of NEUN+ somata within the selected CA1 area. For each mouse, the mean fluorescence intensity was calculated in the contralateral and ipsilateral CA1 area and expressed as percentage of the contralateral value (as no neuron loss is observed in the contralateral hippocampus in the MTLE-HS model). For each group of mice, values are expressed as mean percentages ± SEM (Figure S6C). Statistical analysis was performed using a two-tailed Mann-Whitney test.

## Data Availability

This study did not generate datasets/code.

## Supplemental information

Document S1. Figures S1–S6

Document S2. Article plus supplemental information
