# Supplementary material for: Reprogramming reactive glia into interneurons reduces chronic seizure activity in a mouse model of mesial temporal lobe epilepsy
Source: Cell Stem Cell. 2021 Dec 2;28(12):2104–2121.e10. doi: 10.1016/j.stem.2021.09.002 (PMC8657801; doi:10.1016/j.stem.2021.09.002)
Supplement: Document S1. Figures S1–S6 [file mmc1.pdf]

Cell Stem Cell, Volume *28*

Supplemental Information

Reprogramming reactive glia into interneurons reduces chronic seizure activity in a mouse model of mesial temporal lobe epilepsy

Célia Lentini, Marie d'Orange, Nicolás Marichal, Marie-Madeleine Trottmann, Rory Vignoles, Louis Foucault, Charlotte Verrier, Céline Massera, Olivier Raineteau, Karl-Klaus Conzelmann, Sylvie Rival-Gervier, Antoine Depaulis, Benedikt Berninger, and Christophe Heinrich

FIGURE S1

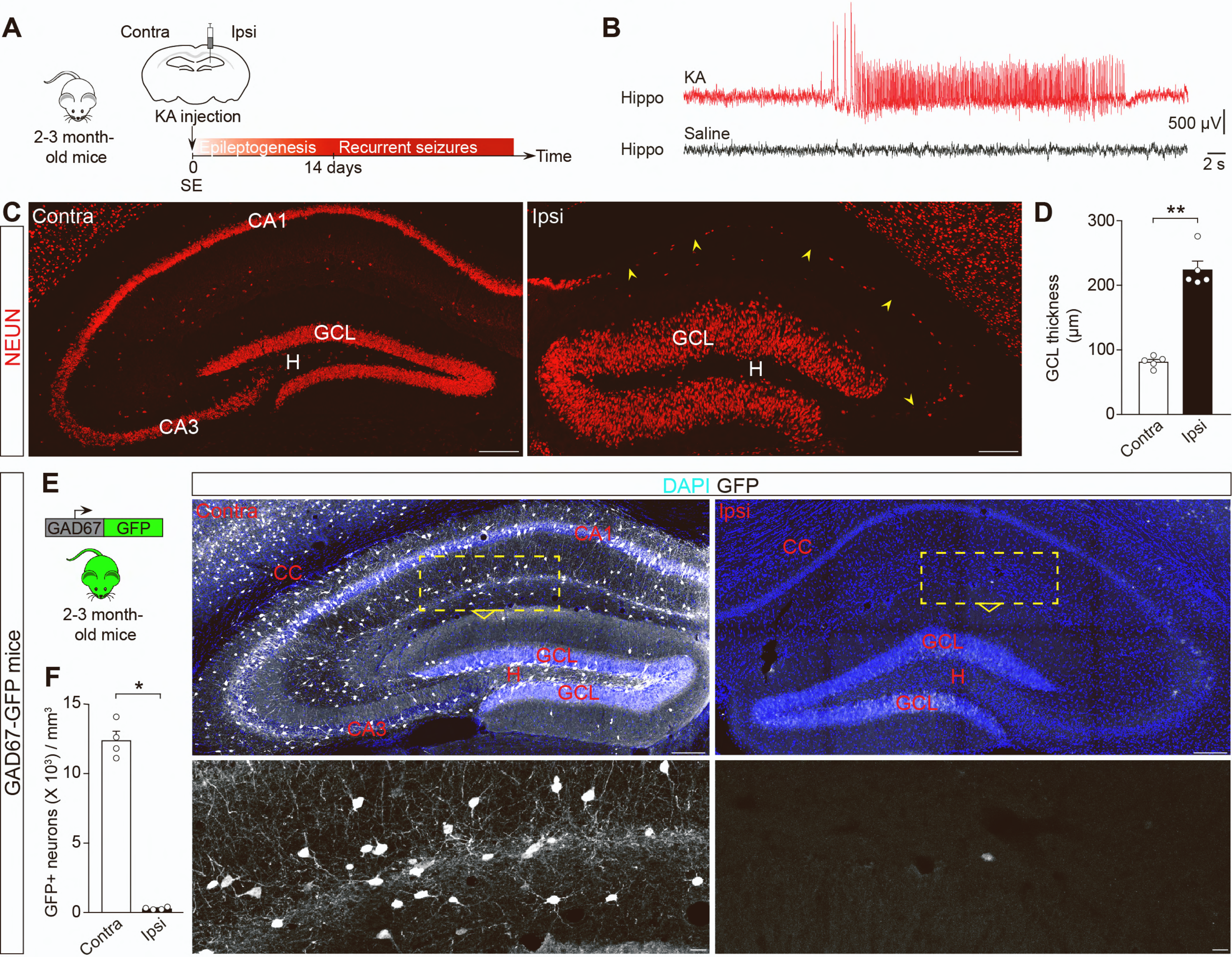
A
Contra
Ipsi
KA injection
2-3 month-old mice
Epileptogenesis
Recurrent seizures
Time
0
SE
14 days
B
KA
Hippo
Saline
Hippo
500 μV
2 s
C
NEUN
Contra
CA1
GCL
H
CA3
Ipsi
GCL
H
D
**
GCL thickness (μm)
300
200
100
0
Contra
Ipsi
E
GAD67-GFP mice
GAD67
GFP
2-3 month-old mice
DAPI GFP
Contra
CA1
CC
GCL
H
GCL
CA3
Ipsi
CC
GCL
H
GCL
F
*
GFP+ neurons (X 10³) / mm³
15
10
5
0
Contra
Ipsi

Figure S1. The MTLE-HS mouse model: Chronic seizure activity, hippocampal sclerosis and loss of GABAergic interneurons in the KA-injected hippocampus (Related to Figure 1)

(**A**) Unilateral intrahippocampal injection of KA initially triggers a SE followed by progressive development within 2 weeks of spontaneous recurrent seizures (i.e., epileptogenesis) that repeatedly occur in the injected hippocampus during the chronic phase of the disease.
(**B**) Representative EEG recording from a MTLE-HS mouse (red) showing a typical non-convulsive EEG seizure recorded in the KA-injected hippocampus during the chronic phase, and consisting of slow rhythmic high-voltage sharp waves followed by higher-frequency and lower-amplitude spikes. EEG recording from a saline-injected control mouse is shown in black.
(**C** and **D**) **Hippocampal sclerosis following KA injection**.
(**C**) NEUN immunostaining depicting extensive loss of neurons within the hilus and all CA hippocampal subfields (arrowheads) as well as pronounced GC dispersion in the KA-injected hippocampus (right) compared to the contralateral (non-injected) side (left).
(**D**) GC layer thickness in the KA-injected hippocampus (black) compared to the contralateral side (white) (n=5).
(**E** and **F**) **Loss of endogenous GABAergic interneurons in the KA-injected hippocampus.**
(**E**) Severe loss of GFP+ (white) GABAergic interneurons all over the KA-injected hippocampus (right) in GAD67-GFP mice, in contrast to the contralateral hippocampus (left), 5 dpKA.
(**F**) Number of GFP+ GABAergic interneurons in the contralateral (white) and KA-injected hippocampus (black) at 5 dpKA in GAD67-GFP mice (n=4).
Bars, mean ± SEM. Statistical analysis in (D) and (F): two-tailed Mann-Whitney test. *$p<0.05$, **$p<0.01$.
Bottom panels in (E): magnified views of boxed areas. Composite images, (C) and (E top).
Scale bars: 200 µm (C, E top), 25 µm (E bottom). GCL, granule cell layer; H, hilus; hippo, hippocampus.

FIGURE S2

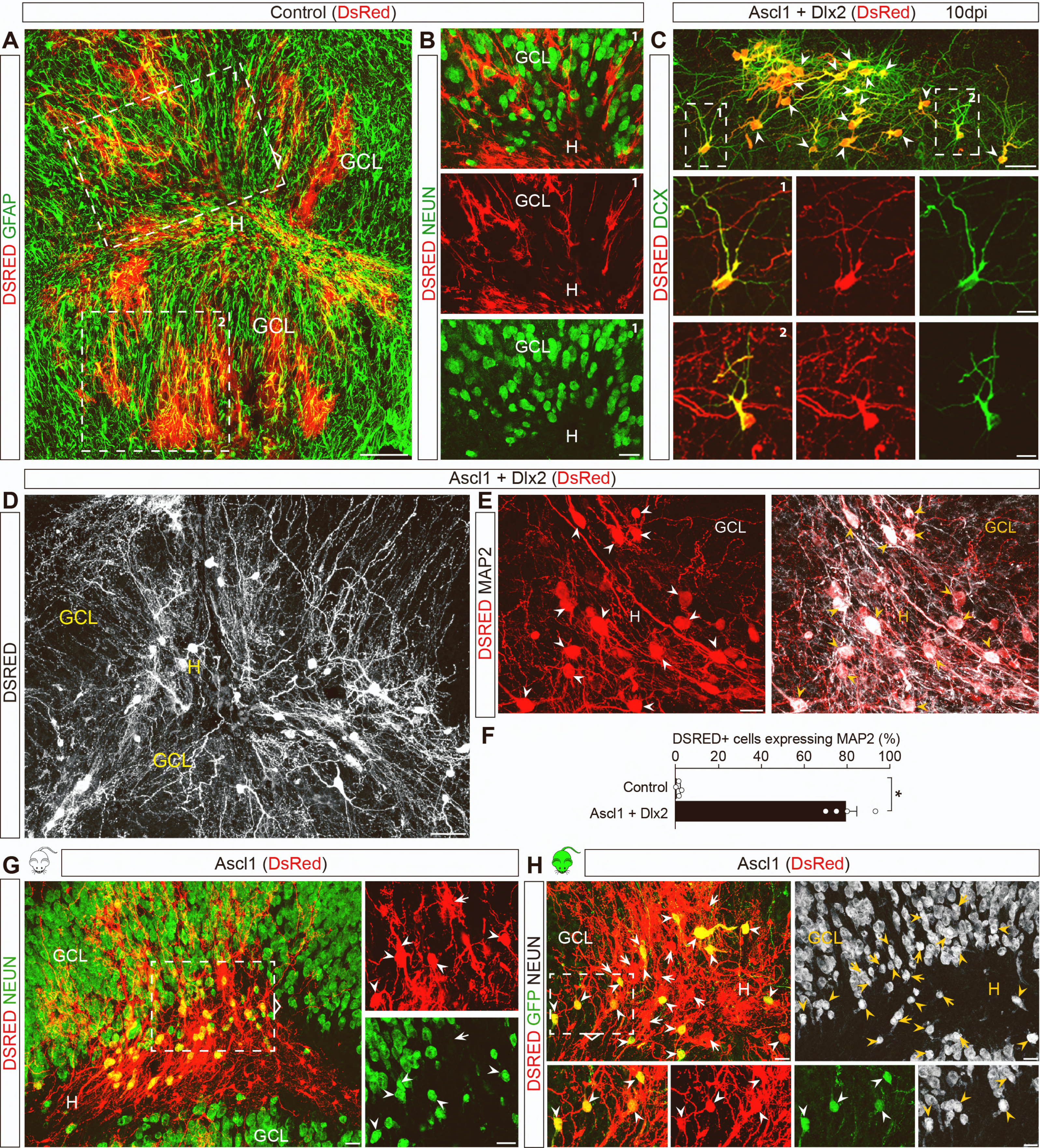

Control (DsRed)
Ascl1 + Dlx2 (DsRed) 10dpi
A
B
C
DSRED GFAP
DSRED NEUN
DSRED DCX
GCL
H
D
E
F
DSRED
DSRED MAP2
Ascl1 + Dlx2 (DsRed)
DSRED+ cells expressing MAP2 (%)
0 20 40 60 80 100
Control
Ascl1 + Dlx2
*
G
H
Ascl1 (DsRed)
DSRED NEUN
DSRED GFP NEUN

Figure S2. *In vivo* reprogramming of grafted cortical astroglia into GABAergic iNs within the MTLE-HS mouse hippocampus (Related to Figure 1)

(**A** and **B**) DSRED+ grafted astroglia transduced with the control retrovirus (DsRed) exhibit astrocyte morphology and express GFAP (A), 2 mpi. None of the DSRED+ cells express NEUN (B; high-magnification of the boxed area #1 in A). Figure 1B shows magnified views of the boxed area #2 in (A).
(**C**) After grafting in the MTLE-HS mouse dentate gyrus, the vast majority of astroglia expressing Ascl1/Dlx2 (DSRED) are reprogrammed into iNs expressing DCX (arrowheads), 10 dpi.
(**D**) Micrograph showing Ascl1/Dlx2-iNs (DSRED, white) exhibiting complex neuronal morphologies and extending processes throughout the KA-injected dentate gyrus, creating dense fiber networks (Same field of view of the dentate gyrus as depicted in Figure 1E).
(**E**) Astroglia expressing Ascl1/Dlx2 are converted into DSRED+ iNs showing MAP2+ dendrites (white, arrowheads), 2 mpi.
(**F**) Proportion of DSRED+ cells converted into MAP2+ iNs following expression of Ascl1/Dlx2 (n=4) or DsRed-only (control, n=4), 2 mpi. Bars, mean ± SEM. Statistical analysis: two-tailed Mann-Whitney test. *$p<0.05$.
(**G**) A substantial number of Ascl1-only (DsRed) transduced astroglia are reprogrammed into DSRED+ iNs expressing NEUN (arrowheads), 2 mpi. The arrow depicts a NEUN-negative DSRED+ glial cell failing to reprogram and exhibiting glial morphology.
(**H**) Ascl1-expressing astroglia derived from GAD67-GFP mice are converted into DSRED/NEUN+ iNs expressing GFP (arrowheads) at 2 mpi, demonstrating their GABAergic identity. Arrows point to DSRED/NEUN+ iNs that do not express GFP.
Bottom panels in (C) and (H) and right panels in (G): magnified views of boxed areas. Composite images, (A), (D), (G) and (H).
Scale bars: 50 µm (A, D), 25 µm (B, C, G, H), 10 µm (C bottom). GCL, granule cell layer; H, hilus.

FIGURE S3

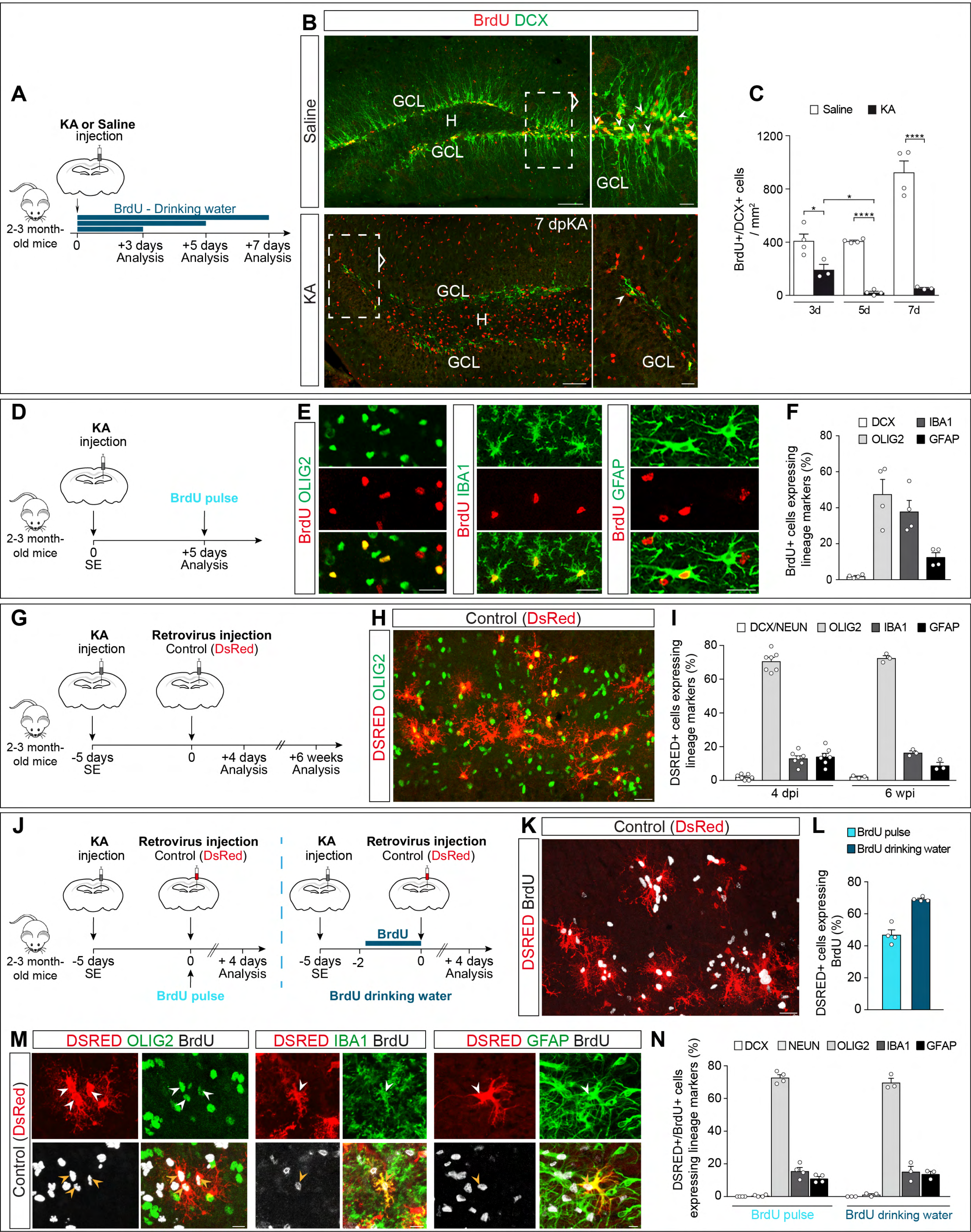

A
KA or Saline injection
BrdU - Drinking water
2-3 month-old mice
0
+3 days Analysis
+5 days Analysis
+7 days Analysis
B
BrdU DCX
Saline
KA
GCL
H
7 dpKA
C
Saline
KA
BrdU+/DCX+ cells / mm²
1200
800
400
0
3d
5d
7d
D
KA injection
BrdU pulse
2-3 month-old mice
0 SE
+5 days Analysis
E
BrdU OLIG2
BrdU IBA1
BrdU GFAP
F
DCX
OLIG2
IBA1
GFAP
BrdU+ cells expressing lineage markers (%)
80
60
40
20
0
G
KA injection
Retrovirus injection Control (DsRed)
2-3 month-old mice
-5 days SE
0
+4 days Analysis
+6 weeks Analysis
H
Control (DsRed)
DSRED OLIG2
I
DCX/NEUN
OLIG2
IBA1
GFAP
DSRED+ cells expressing lineage markers (%)
4 dpi
6 wpi
J
KA injection
Retrovirus injection Control (DsRed)
2-3 month-old mice
-5 days SE
0
+ 4 days Analysis
BrdU pulse
BrdU
-2
BrdU drinking water
K
Control (DsRed)
DSRED BrdU
L
BrdU pulse
BrdU drinking water
DSRED+ cells expressing BrdU (%)
M
DSRED OLIG2 BrdU
DSRED IBA1 BrdU
DSRED GFAP BrdU
Control (DsRed)
N
DCX
NEUN
OLIG2
IBA1
GFAP
DSRED+/BrdU+ cells expressing lineage markers (%)
BrdU pulse
BrdU drinking water

Figure S3. Retroviruses target reactive glial cells, but not neuronal-restricted progenitors, in the MTLE-HS hippocampus (Related to Figure 2)

(**A-C**) **Cessation of adult dentate neurogenesis after KA injection**.
(**A**) Experimental procedures.
(**B**) Dentate gyrus 7 days post saline (top) or KA (bottom) injection and after 7-day BrdU treatment. *Top*, Several BrdU/DCX double-positive cells (arrowheads) are observed in the subgranular zone. *Bottom*, Virtually no BrdU/DCX double-positive cells can be detected at 7 dpKA in line with dramatic reduction in DCX expression.
(**C**) Number of BrdU/DCX+ cells in the subgranular zone at 3, 5, and 7 days after saline (n=4 each) or KA injection (3d, 7d, n=3; 5d, n=4).
(**D-F**) **Massive proliferation of reactive glial cells in the KA-injected hippocampus at the time of retrovirus injection (5 dpKA)**.
(**D**) Experimental procedures.
(**E**) Examples of BrdU+ cells expressing OLIG2 (left), IBA1 (middle) or GFAP (right).
(**F**) Proportion of BrdU+ cells expressing DCX, OLIG2, IBA1, or GFAP in the dentate gyrus at 5 dpKA (n=4).
(**G-I**) **Reactive glial cells proliferating in the KA-injected hippocampus are the only cell types transduced by a retrovirus**.
(**G**) Experimental procedures: Mice were sacrificed at 4 dpi or 6 wpi after control retrovirus (DsRed) injection at 5 dpKA.
(**H**) DSRED+ transduced glial cells, the majority of which express OLIG2 at 4 dpi.
(**I**) Proportion of DSRED+ cells expressing DCX/NEUN, OLIG2, IBA1 or GFAP at 4 dpi (left bars, n=7) and 6 wpi (right bars, n=3).
(**J-N**) **Combining BrdU and retroviral injections results in co-labeling of the proliferating hippocampal reactive glia**.
(**J**) Experimental procedures: Mice were injected with a control retrovirus (DsRed) at 5 dpKA and received either a short BrdU pulse at the same time (*left*) or BrdU in drinking water during a 2-day time-window (*right*) and were analyzed at 4 dpi.
(**K**) The vast majority of DSRED+ cells incorporated BrdU (drinking water protocol).
(**L**) Proportion of DSRED+ transduced cells immunoreactive for BrdU after short BrdU pulse (n=4) or BrdU in drinking water (n=4). Note the high-rate of co-labeling of dividing cells after short BrdU pulse (47%), which was strongly increased when BrdU was supplied in drinking water prior to retrovirus injection (70%).
(**M**) Examples of DSRED/BrdU+ glial cells expressing OLIG2 (left), IBA1 (middle) or GFAP (right)(arrowheads).
(**N**) Proportion of DSRED/BrdU+ cells expressing DCX, NEUN, OLIG2, IBA1, or GFAP after short BrdU pulse (left bars, n=4) or 2-day BrdU in drinking water (right bars, n=3). Marker expression of DSRED/BrdU+ cells was similar following both BrdU protocols, and similar to marker expression of DSRED+ transduced cells (I) and BrdU+ cells (F). No DSRED/BrdU+ cells expressed the neuronal markers DCX or NEUN.
Bars, mean ± SEM. Statistical analysis in (C): two-way ANOVA followed by Sidak's multiple comparison *post hoc* test ('Treatment', $F_{1,16}$=158.1, p<0.0001; 'Days post injection', $F_{2,16}$=17.3, p=0.0001; 'Interactions', $F_{2,16}$=24.2, p<0.0001). *p<0.05, ****p<0.0001.
Right panels in (B): magnified views of boxed areas. Composite images, (B) and (H).
Scale bars: 100 µm (B left), 25 µm (B right, E, H, K), 10 µm (M). GCL, granule cell layer; H, hilus.

FIGURE S4

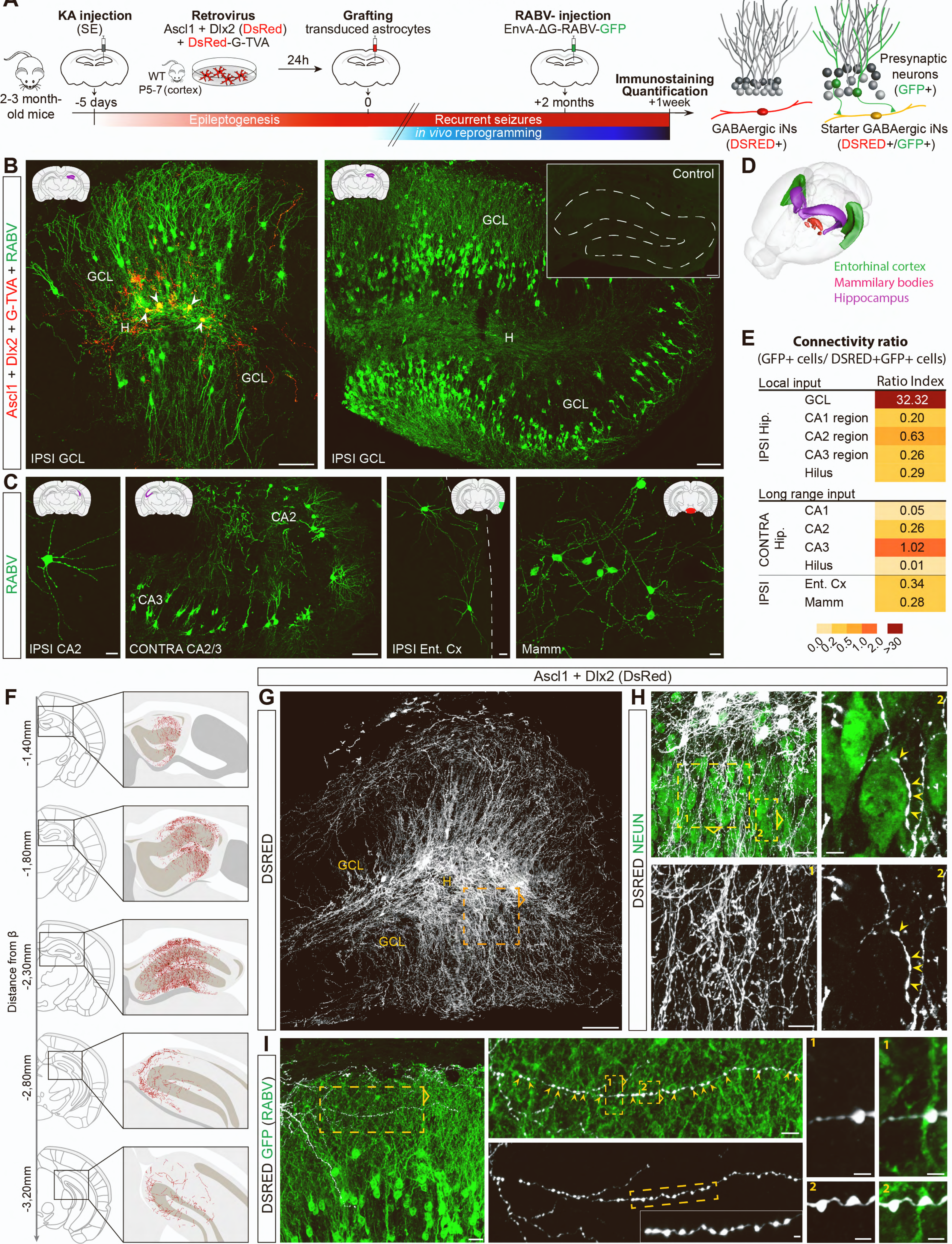
A
KA injection
(SE)
Retrovirus
Ascl1 + Dlx2 (DsRed)
+ DsRed-G-TVA
Grafting
transduced astrocytes
RABV- injection
EnvA-ΔG-RABV-GFP
24h
WT
P5-7 (cortex)
2-3 month-old mice
-5 days
0
+2 months
Immunostaining
Quantification
+1week
Epileptogenesis
Recurrent seizures
in vivo reprogramming
Presynaptic neurons (GFP+)
GABAergic iNs (DSRED+)
Starter GABAergic iNs (DSRED+/GFP+)
B
Ascl1 + Dlx2 + G-TVA + RABV
GCL
H
IPSI GCL
Control
D
Entorhinal cortex
Mammilary bodies
Hippocampus
E
Connectivity ratio
(GFP+ cells/ DSRED+GFP+ cells)
Local input
Ratio Index
IPSI Hip.
GCL 32.32
CA1 region 0.20
CA2 region 0.63
CA3 region 0.26
Hilus 0.29
Long range input
CONTRA Hip.
CA1 0.05
CA2 0.26
CA3 1.02
Hilus 0.01
IPSI
Ent. Cx 0.34
Mamm 0.28
0.0 0.2 0.5 1.0 2.0 >30
C
RABV
CA2
CA3
IPSI CA2
CONTRA CA2/3
IPSI Ent. Cx
Mamm
F
Distance from β
-1,40mm
-1,80mm
-2,30mm
-2,80mm
-3,20mm
Ascl1 + Dlx2 (DsRed)
G
DSRED
GCL
H
H
DSRED NEUN
1
2
I
DSRED GFP (RABV)

Figure S4. iNs derived from grafted astroglia show widespread synaptic integration within the MTLE-HS mouse brain (Related to Figure 3)

(**A**) Experimental procedures.
(**B-E**) **iNs receive local and long-range synaptic innervation from endogenous neurons, 2 mpi**.
(**B**) DSRED/GFP+ starter iNs (left, arrowheads) receive innervation from a massive number of local GFP+ presynaptic GCs in the MTLE-HS dentate gyrus, 2 mpi.
Inset in right panel, No GFP+ cells are visible after RABV injection in absence of primary infection with the retrovirus encoding G/TVA (i.e., in absence of starter neurons), thus ruling out aberrant direct RABV infection of epileptic GCs.
(**C**) Occasional GFP+ presynaptic pyramidal cells are observed in the remaining ipsilateral CA_2 area (Ipsi CA_2). Starter iNs receive innervation from the contralateral hippocampus (Contra $CA_2/_3$) and from long-range projection neurons in the ipsilateral entorhinal cortex (Ent Cx) and mammillary/supramammillary bodies (Mamm). Schematics in (B) and (C) highlight the brain structure shown in each panel.
(**D**) 3D drawing shows brain regions establishing synapses onto iNs.
(**E**) Numbers of GFP+ presynaptic neurons expressed as color-coded connectivity ratios, 2 mpi. (Mean values, n=3).
(**F-I**) **iNs send axonal fibers impinging on dentate GCs, 2-3 mpi** (n=11).
(**F**) DSRED+ fibers from iNs extend over 1.8 mm along the rostro-caudal axis of the dorsal hippocampus in MTLE-HS mice.
(**G**) Ascl1/Dlx2-iNs (DSRED, white) extend fibers forming dense networks throughout the dentate gyrus.
(**H**) *Left panels*, Magnified views of the area boxed in (G) show DSRED+ axons (white) sneaking in between NEUN+ GCs. *Right panels*, Super-resolution images of boxed area #2 depict a DSRED+ axon and axonal swellings (white) in close contact (arrowheads) with a NEUN+ GC soma (single confocal plane is shown).
(**I**) *Left panel,* iNs extend axons (DSRED, white) across dendritic arbors of GCs (GFP from RABV as in B). *Middle and right panels*, Super-resolution images of the successive boxed areas showing numerous *en passant* synaptic bouton-like structures (arrowheads; single confocal planes are shown) contacting successive GFP+ GC dendrites.
Composite images, (B) and (G).
Scale bars: 100 μm (B, C Contra $CA_2/_3$, G), 25 μm (C Ipsi CA_2 - Ipsi Ent Cx - Mamm, H left, I left), 10 μm (H right, I middle), 2 μm (I right). GCL, granule cell layer; H, hilus.

FIGURE S5

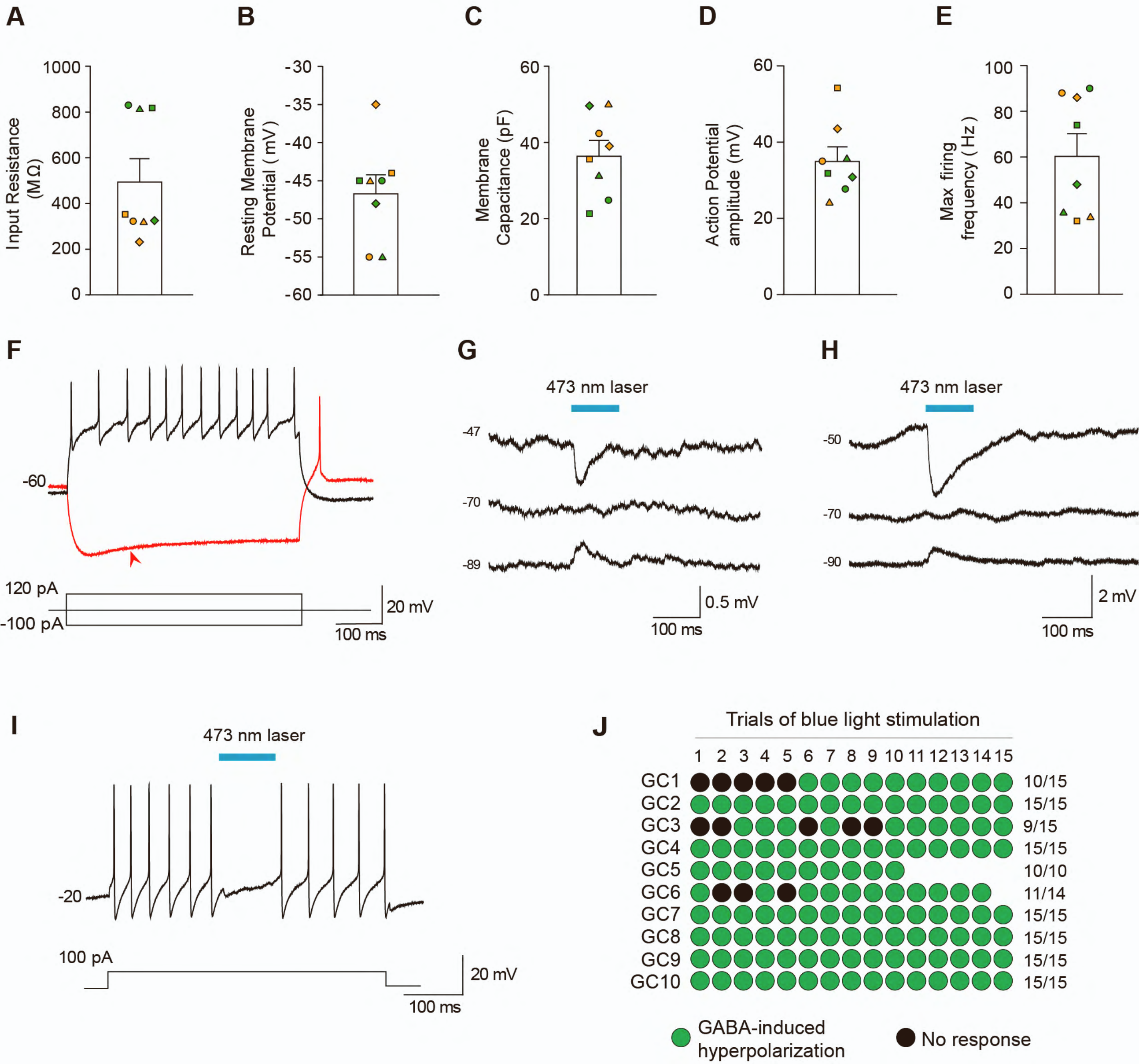

A
Input Resistance (MΩ)
1000
800
600
400
200
0
B
Resting Membrane Potential (mV)
-30
-35
-40
-45
-50
-55
-60
C
Membrane Capacitance (pF)
60
40
20
0
D
Action Potential amplitude (mV)
60
40
20
0
E
Max firing frequency (Hz)
100
80
60
40
20
0
F
-60
120 pA
-100 pA
20 mV
100 ms
G
473 nm laser
-47
-70
-89
0.5 mV
100 ms
H
473 nm laser
-50
-70
-90
2 mV
100 ms
I
473 nm laser
-20
100 pA
20 mV
100 ms
J
Trials of blue light stimulation
1 2 3 4 5 6 7 8 9 10 11 12 13 14 15
GC1 10/15
GC2 15/15
GC3 9/15
GC4 15/15
GC5 10/10
GC6 11/14
GC7 15/15
GC8 15/15
GC9 15/15
GC10 15/15
GABA-induced hyperpolarization
No response

Figure S5. iNs are physiologically functional and form GABAergic synapses onto GCs (Related to Figure 4)

(**A**-**E**) Input resistance (A, MΩ), resting membrane potential (B, mV), membrane capacitance (C, pF), action potential amplitude (D, mV) and maximum firing frequency (E, Hz) of recorded iNs derived from hippocampal glia (yellow, 4 recorded iNs, n=3 mice) or derived from grafted astroglia (green, 4 recorded iNs, n=4 mice). Each iN is identified by the same symbol throughout graphs.
(**F**) Example of a current-clamp recording from an iN showing a time-dependent sag in response to hyperpolarizing current injection (red, arrowhead), a feature reminiscent of low-threshold spiking interneurons.
(**G**-**H**) Responses recorded in GCs at different resting membrane potentials following blue light-mediated activation of CHR2+ iNs derived from hippocampal glia (G) or from grafted astroglia (H). In both cases, the synaptic potentials reverted around the calculated Cl^- reversal potential in our experimental conditions (-70 mV), confirming their GABAergic nature. The upper traces in (G) and (H) are shown in Figure 4 (G) and (N), respectively.
(**I**) Example of a current-clamp recording from a GC surrounded by CHR2+ iN processes and showing transient inhibition of action potential firing in response to blue light-mediated activation of CHR2+ iNs.
(**J**) Diagram showing every individual responses, i.e. GABA-induced hyperpolarization (green) or absence of response (black) in each recorded GC following blue light-mediated activation of CHR2+ iNs derived from hippocampal glia (GC1-4, n=3 mice) or from grafted astroglia (GC5-10, n=5 mice). For each GC, 10-15 consecutive trials were recorded. Note that in both reprogramming paradigms, blue light stimulation of iNs consistently evoked IPSPs in GCs.

FIGURE S6

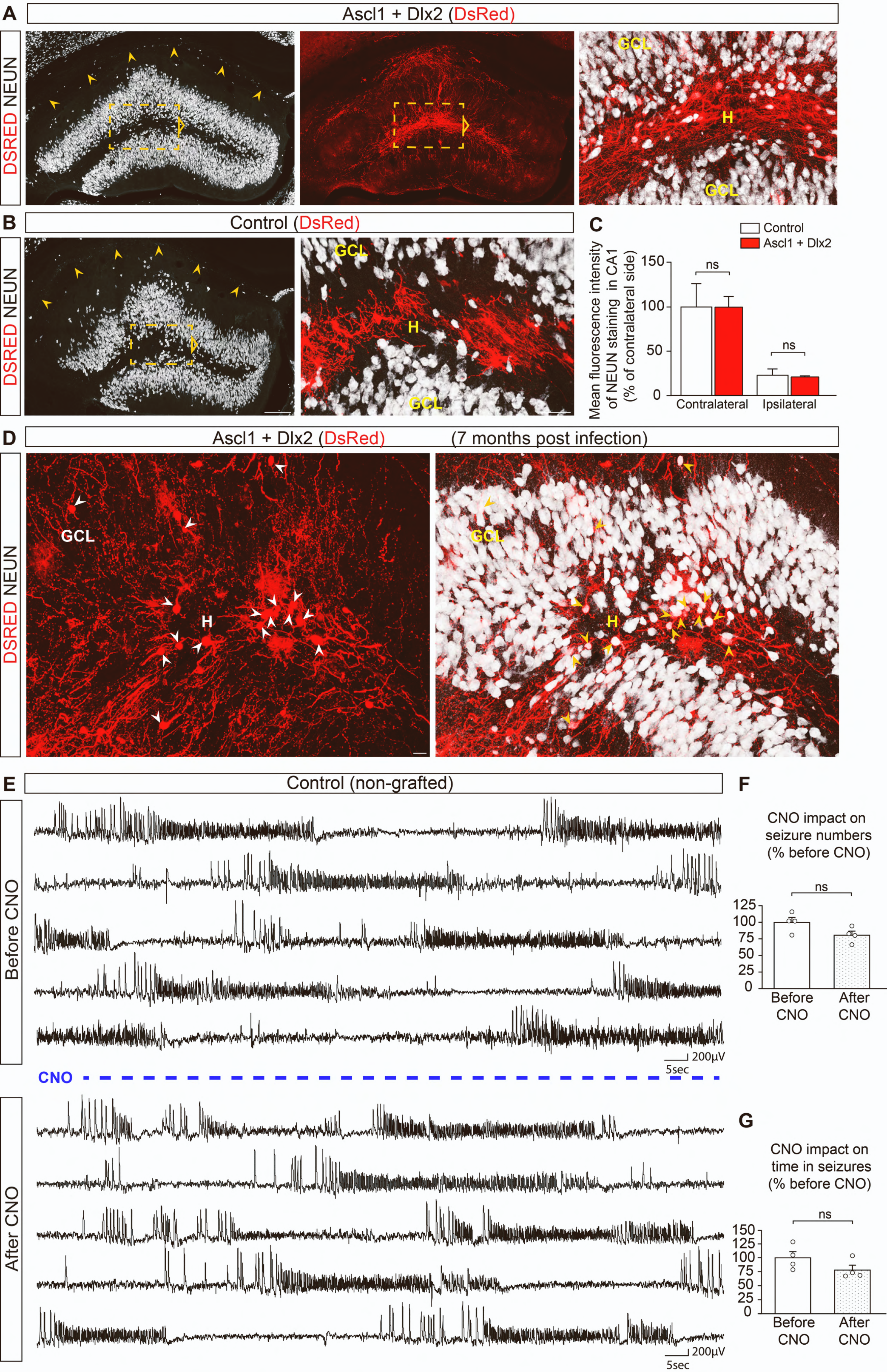

A
Ascl1 + Dlx2 (DsRed)
DSRED NEUN
GCL
H
GCL
B
Control (DsRed)
DSRED NEUN
GCL
H
GCL
C
Control
Ascl1 + Dlx2
Mean fluorescence intensity of NEUN staining in CA1 (% of contralateral side)
150
100
50
0
ns
ns
Contralateral
Ipsilateral
D
Ascl1 + Dlx2 (DsRed) (7 months post infection)
DSRED NEUN
GCL
H
GCL
H
E
Control (non-grafted)
Before CNO
200µV
5sec
CNO
After CNO
200µV
5sec
F
CNO impact on seizure numbers (% before CNO)
ns
125
100
75
50
25
0
Before CNO
After CNO
G
CNO impact on time in seizures (% before CNO)
ns
150
125
100
75
50
25
0
Before CNO
After CNO

Figure S6. Long-term survival of GABAergic iNs in the MTLE-HS mouse hippocampus, and absence of CNO effect on chronic seizure activity in control MTLE-HS mice (Related to Figures 5 and 6)

(**A-D**) **Long-term survival of GABAergic iNs within the sclerotic hippocampus of MTLE-HS mice**.
(**A**) *Left*, NEUN immunostaining at 6 wpi depicts extensive neuronal loss in CA1 (arrowheads) and GC dispersion in EEG-monitored MTLE-HS mice injected with the Ascl1/Dlx2-encoding retrovirus and exhibiting reduced seizure activity (same animals as shown in Figure 5).
Middle, Same field of view as shown in the left panel depicting DSRED+ iNs derived from hippocampal glia and creating dense networks throughout the dentate gyrus.
Right, DSRED+ iNs express NEUN, exhibit complex neuronal morphologies and extend fibers creating dense networks.
(**B**) *Left*, NEUN immunostaining at 6 wpi depicts extensive neuronal loss in CA1 (arrowheads) and GC dispersion in EEG-monitored MTLE-HS mice injected with the control retrovirus (same animals as shown in Figure 5).
Right, DSRED+ hippocampal glia do not express NEUN.
(**C**) Similar extent of neuronal loss in CA1 of the KA-injected hippocampus in Ascl1/Dlx2- and control virus-injected MTLE-HS mice. Bar graph depicts the mean fluorescence intensity of NEUN immunostaining (6 wpi) in CA1 in contralateral (left bars) and KA-injected hippocampus (right bars) in EEG-monitored MTLE-HS mice (shown in Figure 5) injected with the Ascl1/Dlx2-retrovirus (n=6, red bars) or control retrovirus (n=6, white bars).
(**D**) Long-term survival (7 mpi) and integration of iNs (derived from transplanted Ascl1/Dlx2-astroglia) in EEG-monitored MTLE-HS mice showing reduced seizure activity. DSRED+ iNs exhibit elaborated neuronal morphologies, extend fibers forming dense networks throughout the dentate gyrus (left), and express NEUN (arrowheads, right).
(**E-G**) **CNO administration has no significant effects on chronic seizure activity in control MTLE-HS mice**.
(**E**) Representative examples of EEG recordings in the KA-injected hippocampus from a control non-grafted MTLE-HS mouse during the chronic phase, before (top) and after CNO administration (bottom). Note that recordings show similar recurrence of numerous EEG seizures that display a similar pattern before and after CNO treatment. Uninterrupted 10 min intrahippocampal recordings are shown before and after CNO.
(**F** and **G**) Pairwise comparisons of seizure numbers (F) and time in seizures (G) before and after CNO treatment (dotted bars) in control non-grafted MTLE-HS mice (n=4; same mice as shown in Figure 6G, white bars). Comparisons do not reveal significant changes in numbers (F) and cumulative durations (G) of EEG seizures post CNO compared to recordings in absence of CNO.
Bars, mean ± SEM. Statistical analysis: two-tailed Mann-Whitney test (C), and two-tailed Wilcoxon matched-pairs test (F) and (G).
Right panels in (A) and (B): magnified views of boxed areas. Composite images, (A), (B) and (D).
Scale bars: 100 μm (A left - middle, B left, D), 25 μm (A right, B right). GCL, granule cell layer; H, hilus; CNO, clozapine-N-oxide; ns, non-significant.
